# Mangroves in the Galapagos islands: Distribution and dynamics

**DOI:** 10.1371/journal.pone.0209313

**Published:** 2019-01-09

**Authors:** Nicolas Moity, Byron Delgado, Pelayo Salinas-de-León

**Affiliations:** 1 Charles Darwin Research Station, Charles Darwin Foundation, Puerto Ayora, Galapagos Islands, Ecuador; 2 Pristine Seas, National Geographic Society, Washington D.C., United States of America; Fred Hutchinson Cancer Research Center, UNITED STATES

## Abstract

Mangrove forests provide valuable coastal protection from erosion, habitat for terrestrial and marine species, nursery grounds for commercial fisheries and are economically important for tourism. Galapagos’ mangroves usually grow directly on solid lava and fragmented rocky shores, thereby stabilizing the sediment and facilitating colonisation by other plants and many animals. However, until very recently, only inaccurate data described mangrove coverage and its distribution. We mapped mangroves using freely available Google Earth Very High Resolution images based on on-screen classification and compared this method to three semi-automatic classification algorithms. We also analysed mangrove change for the period 2004–2014. We obtained an area of 3657.1 ha of fringing mangrove that covers 35% of the coastline. Eighty percent of mangrove cover is found in Isabela island, and 90% in the western and central south-eastern bioregions. The overall accuracy of mangrove classification was 99.1% with a Kappa coefficient of 0.97 when validated with field data. On-screen digitization was significantly more accurate than other tested methods. From the semi-automated methods, Maximum Likelihood Classification with prior land-sea segmentation yielded the best results. During the 2004–2014 period, mangrove coverage increased 24% mainly by expansion of existing mangroves patches as opposed to generation of new patches. We estimate that mangrove cover and growth are inversely proportional to the geological age of the islands. However, many other factors like nutrients, currents or wave exposure protection might explain this pattern. The precise localization of mangrove cover across the Galapagos islands now enables documenting whether it is changing over time.

## 1. Introduction

### 1.1. Characteristics and importance of mangrove ecosystems

Mangrove forests constitute an assemblage of tropical and subtropical woody trees and shrubs that tolerate high salinity levels (from 0 to ~50 Practical Salinity Units, PSU), allowing them to grow at the interface of marine and terrestrial systems [1,2]. Mangroves inhabit areas commonly flooded by tides with little wave influence, such as shores of sandy soil, clay or silt bays, coastal lagoons, tidal channels (streams), river deltas, low lying sand bars or marshlands. Mangroves build communities parallel to the shoreline that can range from narrow strips a few meters wide, to dense forests that extend across hundreds of hectares inland [2]. Consequently, the distribution of mangroves will depend in great measure on the range of tides, the topographic slope, water salinity, as well as the soil type and its properties [3].

Despite their low species richness, with only 73 species/subspecies identified globally [4] and covering just 0.12% of the Earth’s terrestrial surface [5], mangroves are one of the most productive forest ecosystems on Earth [6]. In addition, mangroves hold significant economic value, providing at least US$1.6 billion per year in ecosystem services worldwide [7], such as carbon sequestration; habitat for terrestrial species; habitat and nursery grounds for socio-economically important marine species; scenic value; or coastal protection against climate-meteorological and hydrodynamic changes [8–14]. While widely recognized for their importance, mangrove forest destruction continues to be a global concern making mangroves one of the most threatened and vulnerable ecosystems worldwide [15]. Mangrove forests are disappearing worldwide at alarming rates and are expected to functionally disappear in 100 years, so while we may not lose every mangrove tree, mangroves will be so greatly reduced that the overall ecological purpose of the ecosystem will be lost [16,17]. Mangrove ecosystem loss is mainly attributed to mangrove forest clearing for shrimp and fish farming, fuelwood, creation of timber, agriculture, coastal development, pollution, and tourism [17]. Ecuador mainland already lost ~40% of the mangrove coverage in the last 40 years due to clearing for shrimp aquaculture [18].

### 1.2. Remote Sensing of mangrove ecosystems

Mangrove ecosystems usually cover large areas of the coastline of tropical and subtropical areas and their mapping and monitoring poses a series of problems, namely, the usually remoteness of the areas where they are found, the logistical complexity of monitoring an ecosystem that is in the sea-land interphase and the vastness of the habitat [2]. Some studies have approached these with surveys from the sea, using motor boats and covering the extension of the ecosystem [19]. However, most of the studies opt to map and monitor mangrove forests with remote sensing techniques through aerial photography or satellite imagery in combination with Geographical Information Systems (GIS) [20,21]. Remote sensing programs are widely used for mangrove mapping because they offer many advantages over other techniques. They allow to detect, identify, map and evaluate mangrove habitat, the monitoring of vast remote tropical and inaccessible areas at habitat or ecosystem scales, rapid and cost-effective data collection, and replicability over time, which is important to monitor changes over time [19,22].

Depending on the purpose of the study and the extent of the mapping exercise, i.e. at landscape, local, regional, continental and global scales, different remote sensing platforms have been used. For estimating global to regional mangrove distributions, medium-resolution programs, like the Landsat series, SPOT (Satellite Pour l'Observation de la Terre) or Sentinel are preferred, see for example [23,24]. For regional to local scales, high-resolution programs are needed, like IKONOS, QuickBird, etc. [25,26] or images from aerial photography, which are usually the only imagery source for retrospective research (starting at ~1940 while the first Landsat image was obtained in 1972) [27] or when very high resolution or high temporal scale is needed [28]. Other remote sensing techniques have also been used for different purposes like Synthetic Aperture Radar (SAR) and Light detection and ranging (LiDAR) technology for estimating mangrove height and biomass [23,29–32].

### 1.3. Mangroves in the Galapagos

Mangrove forests in the Galapagos consist of three species belonging to three families [33]: *Rhizophora mangle* (red mangrove, Fam. Rhizophoraceae), *Avicennia germinans* (black mangrove, Fam. Acanthaceae) and *Laguncularia racemosa* (white mangrove, Fam. Combretaceae). *Conocarpus erectus* (button or buttonwood mangrove, Fam. Combretaceae), is considered a mangrove associate and is found in the transition between true mangrove forests and the arid zone non-mangrove species along with other species like *Hippomane mancinella* (manchineel or poison apple) and *Cryptocarpus pyriformis* (salt bush) [34].

Mangroves in the Galapagos are largely remote from human impacts, they have practically no litter and there is no evidence of mangroves being cut except in the inhabited harbours. However, the Galapagos coastline is pretty inhospitable to mangroves, which partially explains why in many places mangroves are so undeveloped. Indeed, most of the coastline is rocky and exposed to wave activity, with no permanent rivers nor estuaries and with an abrupt coastal topography that narrows the intertidal zone, all factors affecting mangrove establishment [35,36]. On the younger islands, mangroves grow directly on lava fields, constituting narrow bands of lush vegetation abutting barren lava and acting as pioneer vegetation [34,37,38]. On the other hand, in few very enclosed bays protected from the wave energy, mangrove forests are tall, lush and extremely well developed, with trees more than 25 m high in some bays of western Isabela (Wium-Andersen & Hamann 1986). Most probably, the variation of mangrove development around the islands is dependent on how suitable the natural conditions are for their development [34,35,39] as opposed to local human impacts.

Mangroves represent an important ecosystem along the coastline of the Galapagos, providing the islands with important ecosystem services, like carbon sequestration and storage, tourism and fishing. Mangroves include the provision of habitat and nursery grounds to several species of fish, like sharks [40–43], with great importance for the tourism industry [44,45]; or fish with commercial importance, like snappers, mullets, mojarras, milkfish and the Galapagos Sailfin Grouper (*Mycteroperca olfax*) [43,46,47]. Mangrove also constitute an important habitat for terrestrial species. Mangrove forests are particularly important for the critically endangered mangrove finch (*Camarhynchus heliobates*) with only ~100 individuals confined to three small mangrove patches on Isabela Island [48]. Yet, we still lack accurate baseline data quantifying the extent and distribution of mangroves as well as the length of the coastline protected by mangroves. Accurate information on mangrove distribution is of paramount importance as baseline information to study how mangrove coverage is changing over time and the potential effects Climate Change will have locally on this ecosystem. Indeed, mangroves are considered indicator ecosystems of coastal change since they are very sensitive to minor variations on the hydrological regimes [49]. Unlike in Ecuador mainland, where the mangrove forests are already greatly impacted due to clearing for shrimp aquaculture, the Galapagos’ mangroves are close to a pristine state due to the strict protection of the archipelago since 1959, with no shrimp or fish farming industry allowed. Consequently, they provide a living laboratory for the study of global Climate Change threats in a pristine mangrove ecosystem. Likewise, many other studies, like ecosystem services valuation and provision, species conservation and ecological or fisheries studies, need this baseline data. On the other hand, decision-makers and managers of the protected areas of the Galapagos need to know the precise location and extent of this habitat in order to guide the zoning of the protected areas of the archipelago and other management policies, like preserving a certain percentage of the habitat in each bioregion. Hence, it is essential to provide accurate mangrove spatial information for effective conservation planning and decision-making as well as for advancing our scientific knowledge in mangrove-related studies.

### 1.4. Review of mangrove mapping in the Galapagos

The first attempt to map mangrove coverage in the Galapagos archipelago started in 1986 from aerial photographs taken between 1959 and 1960, which estimated 1000 ha of mangrove coverage, although these photographs did not include all the islands of the archipelago [34]. The next attempt to estimate mangrove coverage stemmed from a geomorphology mapping project which relied on Landsat RBV (Return-Beam Vidicon) and MSS (Multispectral Scanner) and aerial photographs taken between 1946 and 1985, and estimated the total mangrove area to be ~2437 ha, but only for the islands of Fernandina and Isabela [50].

The three most recent estimates involved the automated supervised and unsupervised analysis of satellite imagery. The Inventory of the Wetlands of Ecuador project [51] published a mangrove layer for the Galapagos in 2002 (EcoCiencia). This classification used unsupervised classification of Landsat imagery (with a cell resolution of 30 m) complemented with aerial photographs (1:60.000) from the Geographical Military Institute (IGM) and estimated ~3380 ha of mangrove forests. In 2006, the Vegetation Cover and Land Use in the Galapagos Islands project from The Nature Conservancy Centro de Levantamientos Integrados de Recursos Naturales por Sensores Remotos (TNC-CLIRSEN) [52] based their analysis on SPOT satellite imagery (20 m); Landsat when the cloud cover impeded the use of SPOT; and Aster for Genovesa island. The dates of the images ranged from 1999 to 2002, although 80% were from 2000. This study used unsupervised classification, standardized vegetation indices and on-screen visual interpretation in the final step to classify mangrove forests. They estimated an area of 2145.89 ha of mangrove. The most recent study estimating the mangrove coverage in the Galapagos is a revision of native and invasive ecosystem coverage based on a mixed object-oriented classification of 2016 (and 2015 when cloud coverage was problematic) Landsat 8 imagery (15 m resolution panchromatic) yielding a total area of ~1470 ha for the mangrove forest class [53].

Finally, the National Aeronautics and Space Administration (NASA) produced the Global Mangrove Forest Distribution (GMFD), which is the most recent mangrove forests distribution study with a global coverage. They used Landsat imagery (30 m) of 2000 with a hybrid supervised and unsupervised image classification technique [54,55]. They estimated a total of ~2370 ha for the Galapagos islands.

Here, we present an updated report on the mangrove forest distribution at a very fine scale in the protected waters of the most iconic tropical archipelago on the globe using an affordable methodology.

## 2. Methods

### 2.1. Study area

The Galapagos Archipelago is comprised of 18 main islands with more than 100 islets and rocks of volcanic origin about 1000 km off the coast of mainland Ecuador [56]. The islands are emerged peaks of young volcanoes, between 6.0·10^4^ and 5.6·10^6^ years old, representing one of the most volcanically active group of islands in the world [57]. As a result, 46% of the area between the coastline and 1 km inland is made of lava dominated coverage (calculated from [53]), over which mangroves and coastal vegetation have to grow. The Galapagos Islands were declared National Park in 1959 and UNESCO (The United Nations Educational, Scientific and Cultural Organization) World Heritage Area in 1978. The National Park protects 97% of the terrestrial area (~7700 km^2^), the rest of the terrestrial area (3%) is used for human settlements and as productive land (agriculture, livestock, timber and quarries), which occurs on four islands, namely Santa Cruz, San Cristóbal, Isabela and Fernandina. The Galapagos support a human population of ~25 000 residents [58] and 241 700 tourists [59] which provide ~78% of employments [60]. The Galapagos Islands are surrounded by the Galapagos Marine Reserve (GMR), declared in 1998. The GMR is a multiuse protected area which excludes industrial fishing within 40 nautical miles off the archipelago, protecting an area of 138 000 km^2^ and 2 058 km of coastline. Tourism and artisanal fishing are allowed within the GMR which occur at designed places according to the Zoning of the protected areas. The Galapagos islands are well renowned for their extraordinary conservation status, endemism and the very good conservation state of the majority of its ecosystems, which has led to several international recognitions, World Heritage Site, Biosphere Reserve, RAMSAR site, Whale Sanctuary, among others.

**Fig 1:**
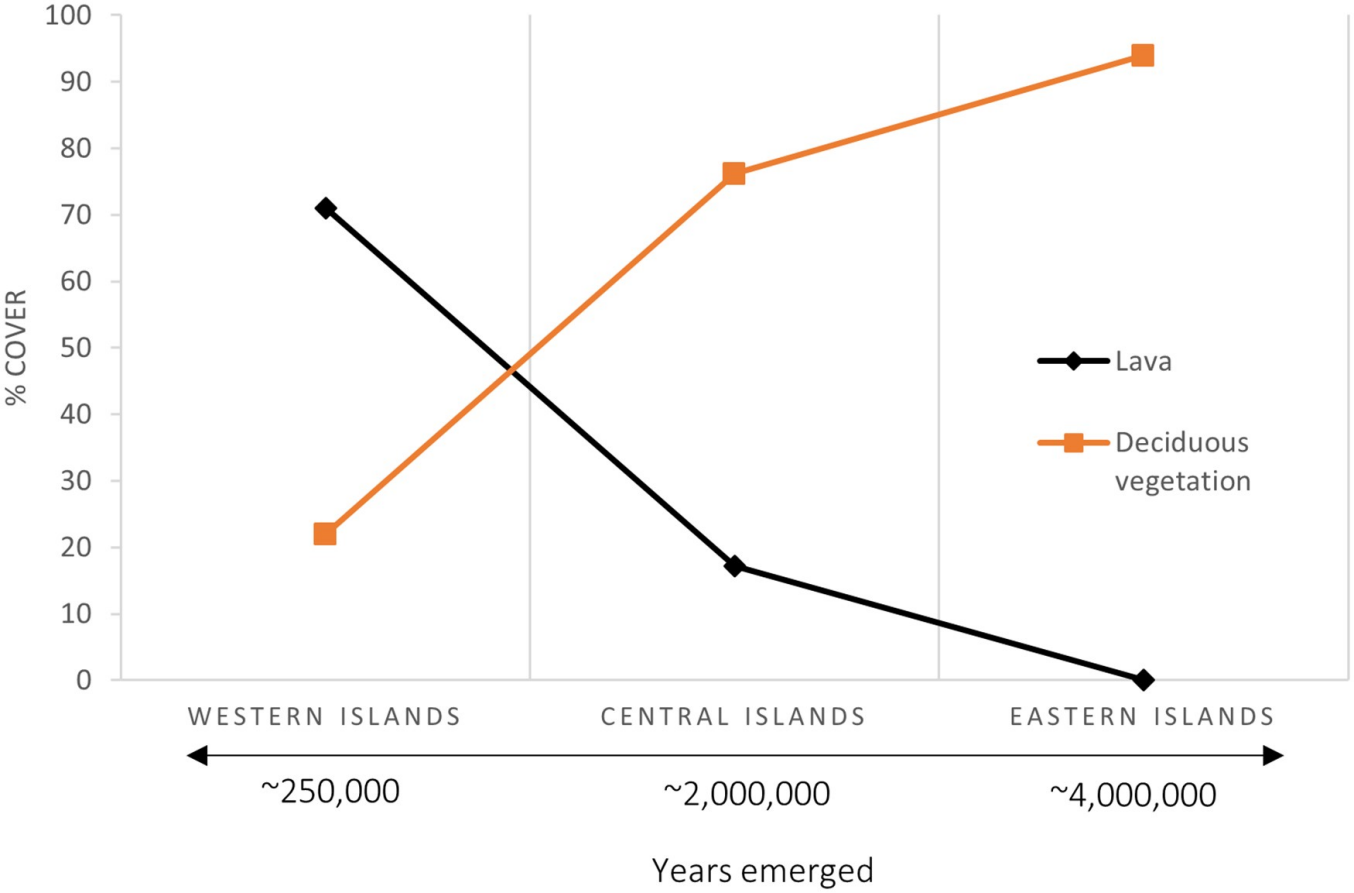
Coastal land-cover (0.5 km from the coastline) with regards to the age of the islands (years emerged averaged from Geist [57]) on the western (Fernandina and Isabela), central (Santiago, Santa Cruz, Rábida, Pinzón, Floreana) and eastern (Santa Fe, Española and San Cristóbal) islands of the Galapagos Archipelago.

The coastal vegetation of the islands is very much dependent on the young volcanic substrate and warm and dry climate [61]. In the coastal area, mean annual precipitation is 469.0 mm, mean annual temperature 24.1 °C and mean annual Sea Surface Temperature (SST) is 23.6 °C (data from the Charles Darwin Research Station, latitude -0.743456°, longitude -90.303710°, at 2 m a.s.l., near Puerto Ayora, 1965–2015 period). According to the Worldwide Bioclimatic Classification System [62], the coastal area of the Galapagos has a tropical xeric bioclimate, with an thermotropical thermotype and semiarid ombrotype. These climate features, in addition to the substrate characteristics, determine in great measure the vegetation composition in the coastal area which has to cope with these harsh environmental conditions [37,61]. As a result, the vegetation of the coastline is pretty scarce (~50% of the coverage is new and old bare lava) and mainly composed of deciduous vegetation (i.e. deciduous forests, shrubland and tallgrass), which make up to ~47% of the cover, the rest being composed of evergreen seasonal forest and shrubland, mangrove forests and coastal humid forest and shrubland (land-cover analysis on the basis of Rivas-Torres et al. [53] and averaged for a coastal area of 0.5–1 km inland from the coastline). Due to the volcanic origin of the islands centred from the Galapagos hotspot, located on the western island of Fernandina [63], there is a west-east gradation of the islands regarding their emergence [57]. This has a direct effect on the coastal vegetation since the youngest islands (the western islands) have a much higher proportion of lava cover and lower of deciduous vegetation while on the oldest islands (the eastern islands) most of the cover is deciduous vegetation, while the lava cover in nearly inexistent (Fig 1).

### 2.2. On-screen digitization

We digitized the extent of mangrove patches directly in Google Earth (GE Pro software v.7.3.2.5487) by tracing the outline of each patch, creating individual polygons by visual photo-interpretation of Google Earth Very High Resolution (GE VHR) imagery. We used visual interpretation and manual delineation of mangrove polygons based on a number of attributes to help delimit mangrove extension. These were primarily based on tonality and texture. Tonality differentiation was used since mangroves usually appear green (from pale green to medium green, depending on the season of the image used) and stand out against the brown-black colour of the lava fields and the green medium mixed with light green or light brown of the deciduous and herbaceous vegetation that can be found next to them. Texture also helps differentiate mangrove cover since mangrove patches present cauliflower patterns clearly distinguishable from the smooth salt marshes usually found in the vicinity. Other attributes, such as shape and closeness to other features, in conjunction with the tonality and texture attributes helped in the interpretation of the images. Mangrove patches frequently show a sinuous perimeter of the assemblage with numerous invaginations and are located in close proximity to water features. We provide a complete list of the attributes we used to interpret the images and differentiate each feature (Table 1). As it has already been demonstrated by [28], the unique combination of tonality, texture and other attributes, made the identification of mangrove features possible. In addition, we used the previous studies on mangrove distribution in the Galapagos (mainly EcoCiencia and the GMFD) to guide our visual interpretation by converting the mangrove polygons from these studies to GE’s native format, KML (Keyhole Markup Language). Since it was impossible to analyse individual trees nor the taxonomic identification of the mangrove assemblages, our analyses were limited to the recognition of species assemblages, i.e. the structure or the tree canopy distribution with respect to each other (as described in [28]). Hence, the maps show the contours of these assemblages. Once digitized, mangrove polygons were saved to KML format in the ETRS (European Terrestrial Reference System) 4326 coordinate system and later transformed to the ESRI (Environmental Systems Research Institute) shapefile format in Quantum GIS [64] and converted to ETRS 32715 in order to conduct further analysis. All recognizable patches were digitized at an eye altitude of 300 m in GE. In some cases, we diminished the eye altitude to 150 m to meticulously check dubious patches. The minimum mapping unit (MMU) used in this study was 10 m^2^.

**Table 1:** Google Earth Very High Resolution image interpretation attributes to identify mangrove patches and distinguish mangrove from other features.

Tonality	Texture	Other attributes	Feature
Green medium	Cauliflower coarse	Individual crowns difficult to distinguish from each other. Frequently sinuous perimeter of the assemblage. Continuous assemblage identified as a unit differentiable from other features. Closeness to water feature (open sea, cove, pond).	Mangrove*
Green pale	Cauliflower fine	Individual crowns easier to distinguish from each other. Frequently sinuous perimeter of the assemblage. Continuous assemblage identified as a unit differentiable from other features. Small shadow. Closeness to water feature (open sea, cove, pond).	Mangrove*
Green medium to dark green	Diffuse cauliflower		Mangrove*
Green medium mixed with green light	Cauliflower coarse	Individual crowns easier to distinguish from each other, light green crowns adjacent to medium green crowns, not continuous assemblage. Medium shadow.	Deciduous vegetation
Brown light	Cauliflower coarser or fine		Deciduous vegetation
Light green	Smooth		Herbaceous vegetation
Green light	Cauliflower coarse		Other coastal vegetation
Green light	Cauliflower fine		Other coastal vegetation
Brown dark with some green	Fine grained		Lava with some vegetation
Brown light	Smooth		Lava with some vegetation
Brown dark	Fine grained		Lava
Dark grey-Black	Fine to coarse		Lava
White-Light yellow	Smooth		Sand
Green pale	Smooth	Water, mangrove cove/pond	Water
Dark green	Smooth	Water, mangrove cove/pond	Water
Pure White	Smooth	Following line patterns, frequently visible wave wake	Breaking waves
Light green-Turquoise	Smooth	Water	Underwater sand
Faded brown-Dark brown	Fine grained	Water	Underwater lava

* It is important to note the season of the images used in the digitization. As mentioned before, even if mangrove vegetation is always green, during the humid season the mangroves appear as strong or live green while in the dry season mangrove color turns to pale green.

#### 2.2.1 Relative scale of the mangrove layer

In order to determine the scale of the mangrove layer produced in this study and make it comparable with previous studies, we applied a method to define the relative scale of the mangrove layer. We compared the fitting of the perimeter of the digitized polygon with the actual mangrove patch in GE VHR imagery. The resulting scale is relative because it depends on the computer screen resolution, however it allows the comparison between this and previous studies. For this calculation, we started from the eye altitude of 300 m in GE and then zoomed in or out until the digitized polygon adjusted perfectly to the mangrove patch in GE. We recorded the eye altitude and transformed it to a relative scale value for each sampled polygon taking into account GE’ scale bar. To sample the polygons, we used a stratified random sampling (depending on the number of polygons per island) of n = 573 polygons (5% margin of error and 99% confidence interval). The resulting relative scale is the mean ±SE (Standard Error) of the sampled polygons.

### 2.3. Remotely sensed datasets

We took advantage of the readily availability of GE VHR imagery to map mangrove forests in most of the archipelago. However, in areas with poor image quality (i.e. low resolution, poor illumination/contrast or presence of clouds), we used Bing Maps imagery instead via its Web Map Service (WMS) in Quantum GIS [64]. Around the inhabited islands of Santa Cruz, Floreana, San Cristóbal and Isabela, GE VHR imagery was complemented with visual analysis of orthophotos from the SIGTIERRAS project of the Ecuadorian Ministry of Agriculture, Livestock, Aquaculture and Fisheries (MAGAP) (Table 2). GE VHR images were acquired between 2005 and 2015, with a majority of the images captured in 2014 (details of the dates of the images per island are available in S1 Table).

**Table 2:** Remotely sensed data sources used in the digitization of mangroves.

Name	Source	Type of source	Cover	Spatial Scale	Temporal Scale
SIGTIERRAS	SIGTIERRAS Project (Ministry of Agriculture, Livestock, Aquaculture and Fishing, Ecuadorian Government)	Orthophoto	Isabela, San Cristóbal, Santa Cruz and Floreana	1:5000	2010
Bing Maps	Microsoft Bing Maps (Microsoft Redmond Campus, Redmond, WA, USA)	Remotely sensed images via WMS (Web Map Service)	All of the Galapagos Archipelago	Variable	July 2004
Google Earth	Google Earth Pro 7.3.1 (Google, Inc. Mountain View, CA, USA)	Remotely sensed images	All of the Galapagos Archipelago	Variable	2005–2015

### 2.4. Mangrove patch complexity

The outer limits of the mangrove patches often appeared very irregular as a result of environmental conditions such as availability of favourable soil, predation, competition with other species, etc. Therefore, the polygons representing such patches should reflect that irregularity. Accordingly, we measured the irregularity of the outer boundaries of the polygons, for which we use the term ‘complexity’ to highlight the difference between the layer obtained in this project and previous studies. Even though humans are able to differentiate a simple polygon from a complex one at first glance, it is difficult to create a metric to measure the complexity of a polygon [65]. This complexity measure was used as a way to measure the agreement between true mangrove outline shape and its representation according to the image classification method.

In this study, we used three measures of complexity to compare the polygons produced in this study with those of previous studies. We determined the first measure of complexity (Complexity 1) by calculating the perimeter/area ratio for each polygon, with high values corresponding to a more complex polygon; that is, the more invaginations and outgrowths of the polygon, the greater the perimeter/area relationship.

Complexity1(pol)=perimeter(pol)area(pol) (1)

Another measure of complexity (Complexity 2) followed a modification of the method described in [65]. This algorithm consists of three parameters: the relative increase of the boundary amplitude *(ampl)*, the number of nodes *(nodes)*, and the convexity of a polygon *(conv)* according to the following equations:

ampl(pol)=perimeter(pol)-perimeter(convexhull(pol))perimeter(pol) (2)

nodes(pol)=totalnodes(pol) (3)

conv(pol)=area(convexhull(pol))-area(pol)area(convexhull(pol)) (4)

A node represents a vertex where there is a change in direction in the perimeter of a polygon. C*onvex hull* refers to the smallest polygon that encompasses all points or nodes of interest. An intuitive way to view the convex hull is to imagine the shape of a rubber band that stretches to enclose all outer nodes of interest.

The relative increase of the boundary amplitude indicates a more complicated object shape relative to the *convex hull*. Given the fact that the mangrove patches are usually sinuous, it was difficult to calculate *frequency sensu* Brinkoff. Therefore, we used the number of nodes as a proxy for frequency. Using the parameters described above and based on Brinkoff`s work, the complexity would be defined as such:

Complexity2(pol)=0.8·ampl(pol)·nodes(pol)+0.2·conv(pol) (5)

**Fig 2:**
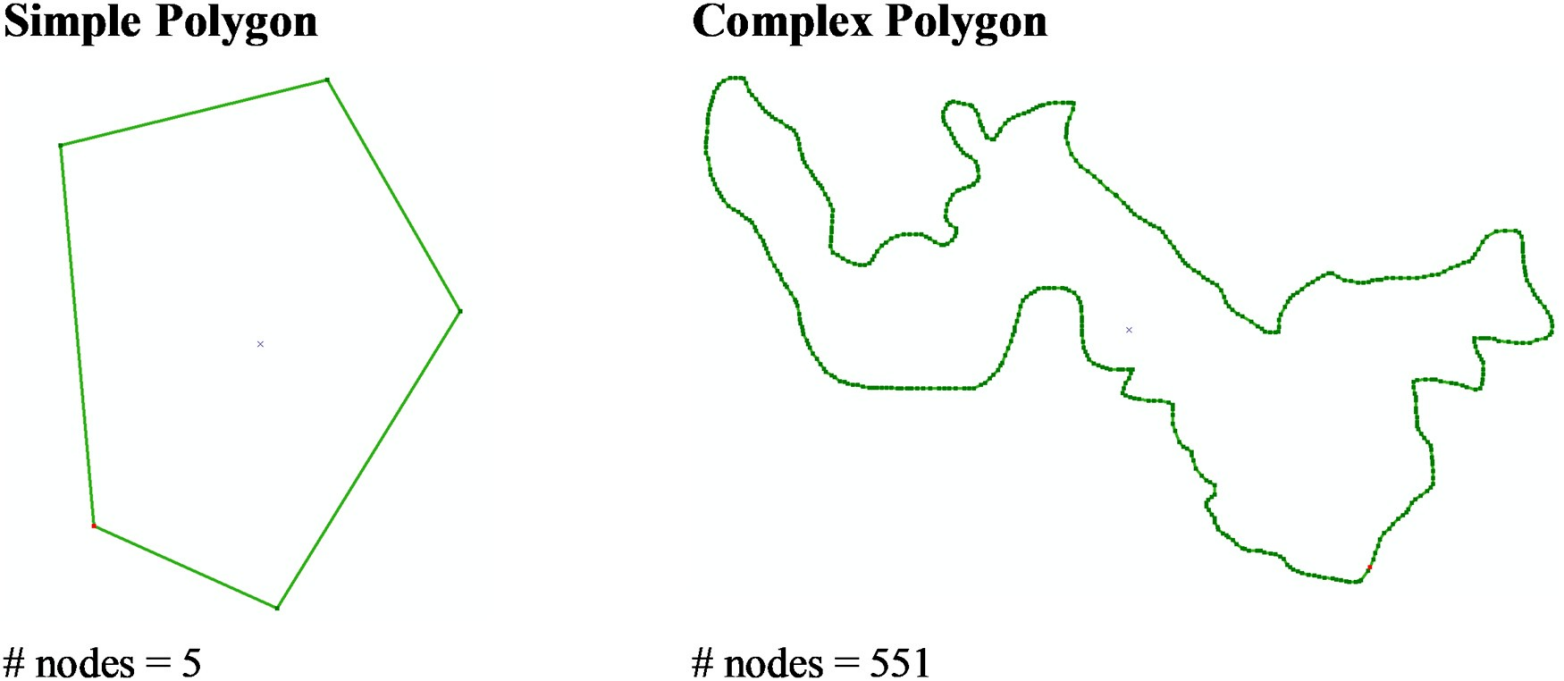
Difference in the number of nodes between a simple and complex polygon.

Finally, the third complexity measure (Complexity 3) calculated the total number of nodes in each polygon. A complex polygon would therefore have more nodes than a simple one (Fig 2).

Complexity3(pol)=∑i=1nnodes(pol)n (6)

*n* represents the number of polygons.

### 2.5. Validation of the classification

#### 2.5.1 Digitizing and classification accuracy

Since the digitizing was performed by three different GIS technicians, we performed a quality check of the mangrove patch digitization process by means of a stratified random sampling of 8% (n = 328) of the total number of polygons (n = 4099). The review process compared the perimeter of the digitized polygon with the actual mangrove patch visualized in GE at an eye altitude of 300 m. A polygon was considered accurate when ≥ 50% of the perimeter of the polygon coincided with the perimeter of the actual patch of mangrove, otherwise it was considered inaccurate.

To evaluate the accuracy of the classification process we used ground-truth data from field trips conducted between 2015–2018, visiting 208 sites across 15 islands, all except Pinta and Rábida. During these trips, we visually verified mangrove presence/absence, and geo-referenced the sites with a handheld Global Position System (GPS) (Garmin GPSMAP 78). Since our data is produced in vector format, we cannot use pixels to evaluate the accuracy. Furthermore, we only vectorised the mangrove cover, not lava fields, other vegetation formations, sand, water, etc. Thus, in order to construct a confusion or error matrix we distinguished between mangrove and non-mangrove classes, and buffered an area of 3.14 ha around each of the field sites (based on our ability to visually estimate the distribution of mangrove and non-mangrove classes within a 100 m radius from the central GPS point taken in the field). We then randomly placed 500 sample points inside the buffer area to evaluate the accuracy of mangrove presence. At each point we verified the presence or absence of mangroves. We compared our classified map against field observation data to determine the overall accuracy of the map, calculating the proportion of correct sample points in relation to the total number of sample points [66]. This procedure was iterated three times to ensure objectivity in the assessment. We also calculated the Kappa statistic which reflects the difference between actual agreement and the agreement expected by chance [67], e.g. a Kappa of 0.95 means the likelihood of agreement is 95% better than by chance alone. In order to compare our results with previous mangrove mapping studies and with other image classification techniques, we used the same procedure to evaluate the overall accuracy and Kappa statistic, but in this case the number of sample points was 100.

Finally, we performed a Z-test between GE-based and the GMFD, EcoCiencia, Rivas-Torres et al. [53] and TNC-CLIRSEN [52] studies to determine the significant differences between the classification processes using the following formula [68]:

Z=X1N1-X2N2ρ(1-ρ)(1N1+1N2) (7)

Where *N*_*1*_ and *N*_*2*_ refer to the total number of sampling points, *X*_*1*_ and *X*_*2*_ the total number of points that were correctly classified and ρ is calculated as (*X*_*1*_ *+ X*_*2*_) / (*N*_*1*_ *+ N*_*2*_). The derived Z value is compared against tabulated Z values at a 5% level of significance to assess if there are statistical significant differences between classification processes. If |Z| is ≥ 1.96, there is a statistically significant difference between the different classification results [69]. We also tested if there were significant differences in accuracy between the three GIS technicians that performed the on-screen digitization.

#### 2.5.2 Semi-automatic classification

**Fig 3:**
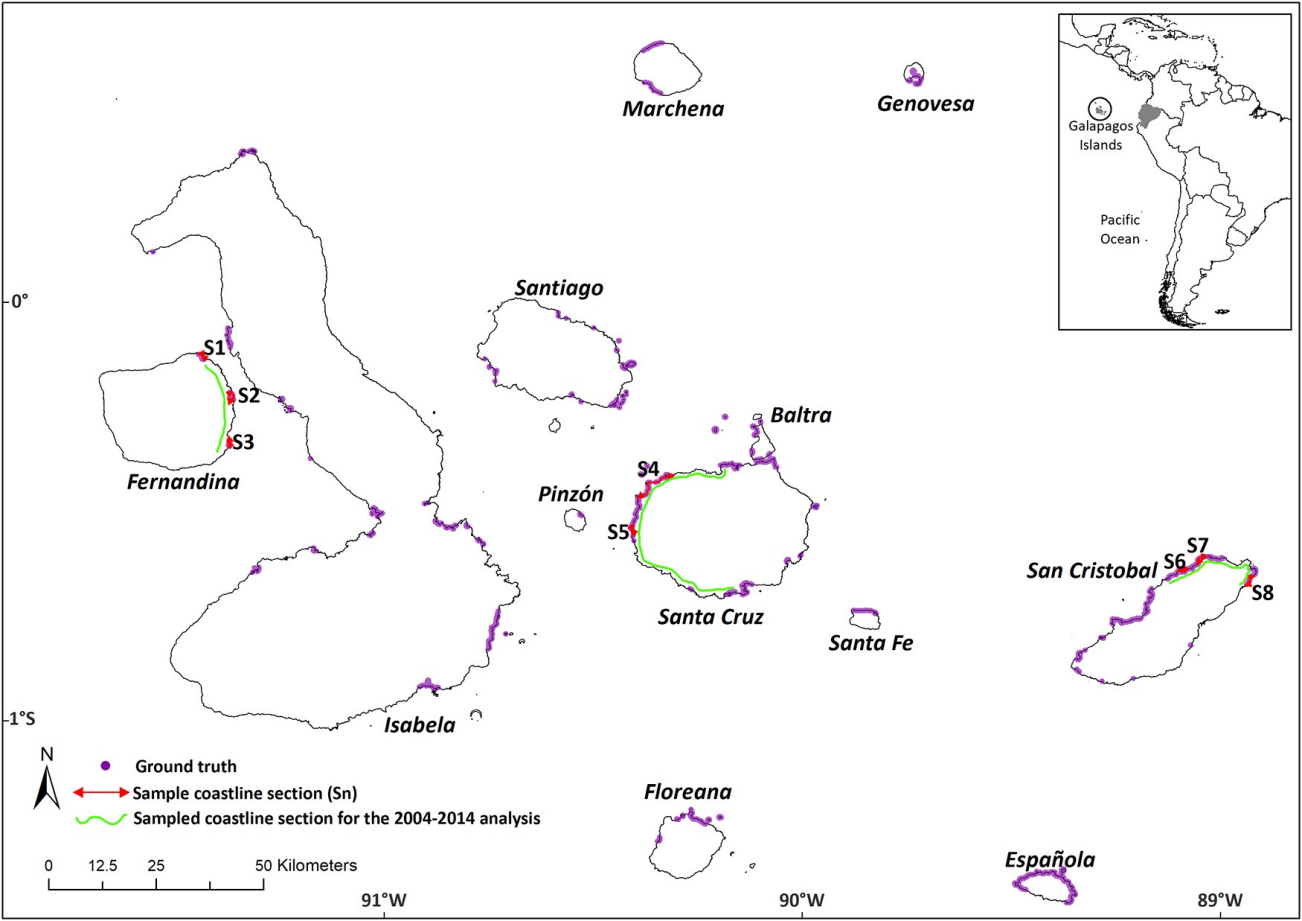
Map of the sampled sections of the coastline to test several semi-automatic classification and hybrid algorithms (red sections) and to perform the temporal 2004–2014 analysis (green sections). Ground truth points are also shown (magenta points).

In order to compare the results and accuracy of our processing method with classic processing chain image classification methods [70], we compared digitization over GE VHR imagery in GE software with two pixel-based semi-automatic classification methods of the same GE imagery. Namely, the Maximum Likelihood Classification algorithm (MLC) and a hybrid method using image segmentation (OBIA, Object Based Image Analysis) with MLC. Software accessibility, free sources of imagery availability, spatial and temporal resolution and computational power, were the factors considered to decide which chain to choose for the comparison. The comparison was performed over some representative coastal sample areas across the archipelago were there was a high density of ground truth points. We chose areas from the east coast of Fernandina (representing a geologically young island), the west coast of Santa Cruz (representing a medium age island) and the northeast coast of San Cristóbal (representing an older island) (Fig 3). We followed the methodological procedure described in Collin et al. [71] with some modifications. Semi-automatic classification procedures need the analysis of satellite imagery locally, in an GIS software. We used the free and open source software SAS Planet 160707 to download and georeference GE imagery from the sample areas. The imagery was downloaded in ECW (Enhanced Compression Wavelet) format, ETRS 4326 reference system, with a zoom level of 20x (equivalent to an eye-altitude of 300 m) and was limited to a one kilometre buffer from the coastline in order to facilitate the classification of mangrove cover. All areas outside the buffer were set to *no data* values to simplify image management and processing. Due to the different sensors that GE VHR imagery mosaic compiles, we split image composite in four sections according to homogeneity criteria and we further analysed those sections for the purposes of image classification at a digital number level (see Fig 3 for the location of the sampling sections). To integrate the data in ESRI’s ArcGIS 10.3 software, the images were transformed to GRID Stack 7.x., and projected to ETRS 32715 to be compatible with ground truth validation data.

The first method, the MLC algorithm (hereafter MLC1), is based on the RGB (red, green and blue) bands of the images, and first needs the digitization of regions of interest (training samples) for each land cover, namely bare ground, water, vegetation, and mangrove [71]. The training samples were simplified with the help of an analysis of the histograms of the digital numbers. For example, the first training sample survey produced 12 water types, which were simplified to five. This process was applied to each homogenous image composite. The training samples were used to feed MLC algorithm in ArcGIS Image Classification tool. The classification raster was converted to polygon features, to obtain the area values of the image classification output.

In order to help with the classification of mangrove forests, we separated the land from water using the coastline polyline. Then, the MLC algorithm was applied over the marine area devoid of land and to the land area devoid of sea, thus, each portion of the image composite was classified at a time. This method (hereafter MLC2) was applied as it has been proved by others to increase the classification accuracy with the MLC algorithm over coastal areas [71].

The third method, hereafter HYBRID, used a hybrid chain. In order to enhance the quality of image analysis and assist the classification of the images, we restrained the object analysis of the images to vegetated areas. To do so, we first differentiated vegetated cover from non-vegetated cover by analysing the multispectral data of Sentinel 2A L1C imagery [72]. Images were corrected atmospherically with Dark Object Subtraction (DOS1) [73] with the help of the Quantum GIS Semi-Automatic Classification plugin. To identify vegetation cover, we calculated the Normalized Difference Vegetation Index (NDVI) which is an index of the absorptive and reflective characteristics of vegetation proportional to the absorption of photosynthetically active radiation [74]. It is calculated from the visible red (R) at and near infra-red (NIR) light (wavelengths of 664.5 nm and 835.1 nm in Sentinel 2A images, respectively) reflected by vegetation as per this formula:

NDVI=NIR(Band8)-R(Band4)NIR(Band8)+R(Band4) (8)

which result in a number that ranges from minus one (-1) to plus one (+1) and reflect the density of green leaves (zero meaning no vegetation). For this study, a threshold of 0.2 was used to differentiate vegetated (≥0.2) from non-vegetated cover (<0.2). This value was determined testing different thresholds in ground-truth areas to determine which was the minimal NDVI value able to differentiate vegetated from non-vegetated areas. All the non-vegetated areas were masked from GE VHR images and the OBIA *i*.*segment* algorithm [75] was applied (*i*.*segment* parameters were set to a difference threshold of 0,75 and a minimum segment size of 750 pixels). Finally, we updated these segmented images by selecting the segments that intersected with the mangrove class obtained in the first chain of the MLC (MLC2) as it has been proved to enhance the OBIA classification results [76].

For the three tested methodologies, a MMU of 10 m^2^ was used, so all polygons smaller than the MMU were erased. Comparisons of the four tested methods between island groups according to their geological age were done using Kruskal-Wallis tests (non-parametric ANOVA) and Dunn test for multiple comparisons.

We also tested two RGB vegetation indices with GE VHR imagery, namely Triangular Greenness Index (TGI) and Visible Atmospheric Resistant Index (VARI) [77,78] with very bad results at vegetation recognition for the sampled areas. Thus additional analysis with these techniques was not further explored.

#### 2.5.3 Temporal change

To examine if the differences in mangrove coverage between years has been a difference due to environmental issues or an artefact from the methodology used in each study, we analysed the mangrove cover ten years back from the present study’s imagery with the history imagery tool available in GE software. The target imagery time was 2004, however, due to image availability over different areas, we had to load imagery between 2004–2007, although most of the images were from 2004. Mangrove cover distribution for 2004 was done in the same way as for the 2014 survey, by means of on-screen digitization in GE software and further transformation from EPSG 4326 to the local reference system EPSG 32715.

For the mangrove cover temporal change we chose three sample areas covering the east coast of Fernandina, the west coast of Santa Cruz and the northeast of San Cristobal so as to cover different bioregions (*sensu* [79]) and geological ages of the islands (Fig 3). We also analysed the mangrove cover for those sampled areas for the previous studies to be able to compare with the mangrove cover from our ten-year span.

To analyse the spatial-temporal changes in mangrove cover for the sampled areas, we first examined the differences in the size frequency distribution of the mangrove patches between the two time periods with Kolmogorov–Smirnov (K-S) tests. For a deeper understanding of mangrove change, i.e. the development of new mangrove patches, the loss of patches and the expansion or contraction of patches already present, we used the geographic technique “spatial–temporal analysis of moving polygons” (STAMP), available through the ‘stampr’ R package [80]. To be able to run the STAMP analysis, 2004’ polygons had to be geographically corrected for the known issues in GE imagery position errors [81]. A 15 m spatial distance threshold was applied to the STAMP analysis and was determined by our MMU and by a brief analysis where we calculated the average distance between 15 pairs of sample points for each time period, 2004 (*t*) and 2014 (*t*_*+10*_), of the same geographical location across the three sampled islands. This was done using abiotic, relatively stable features like capes, big rocks, etc. We used the ESRI’s ArcGIS 10.3 software to visualize STAMP’s four levels of complexity and to calculate summarized statistics of these results by island. All statistical analysis were done using the statistical software R v. 3.5.1 [82].

## 3. Results

### 3.1. Mangrove distribution in the Galapagos

**Fig 4:**
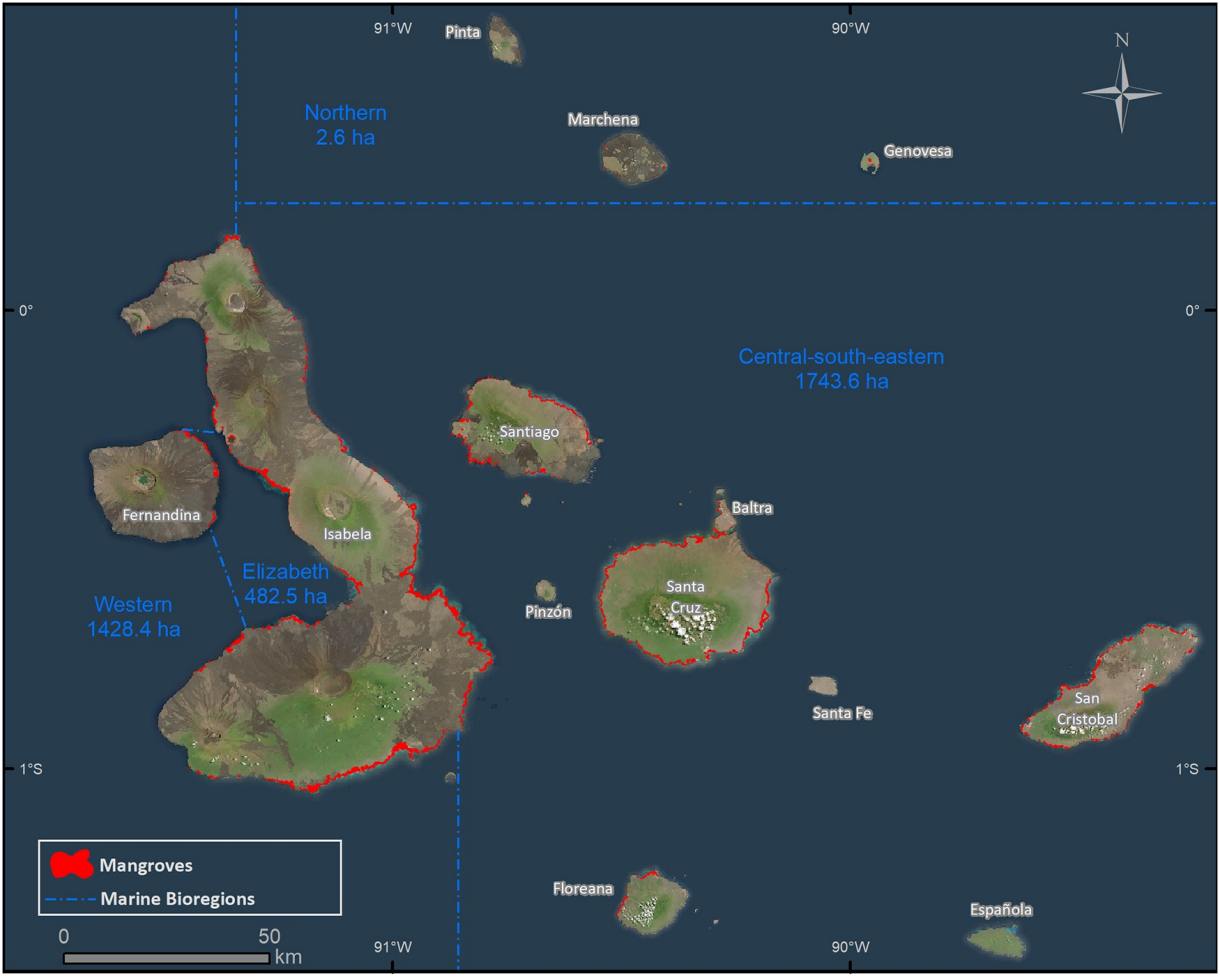
Distribution of mangrove forests in the Galapagos Islands over Landsat 8 imagery composite. Mangrove cover (ha) per bioregion (*sensu* Edgar et al, 2004).

**Fig 5:**
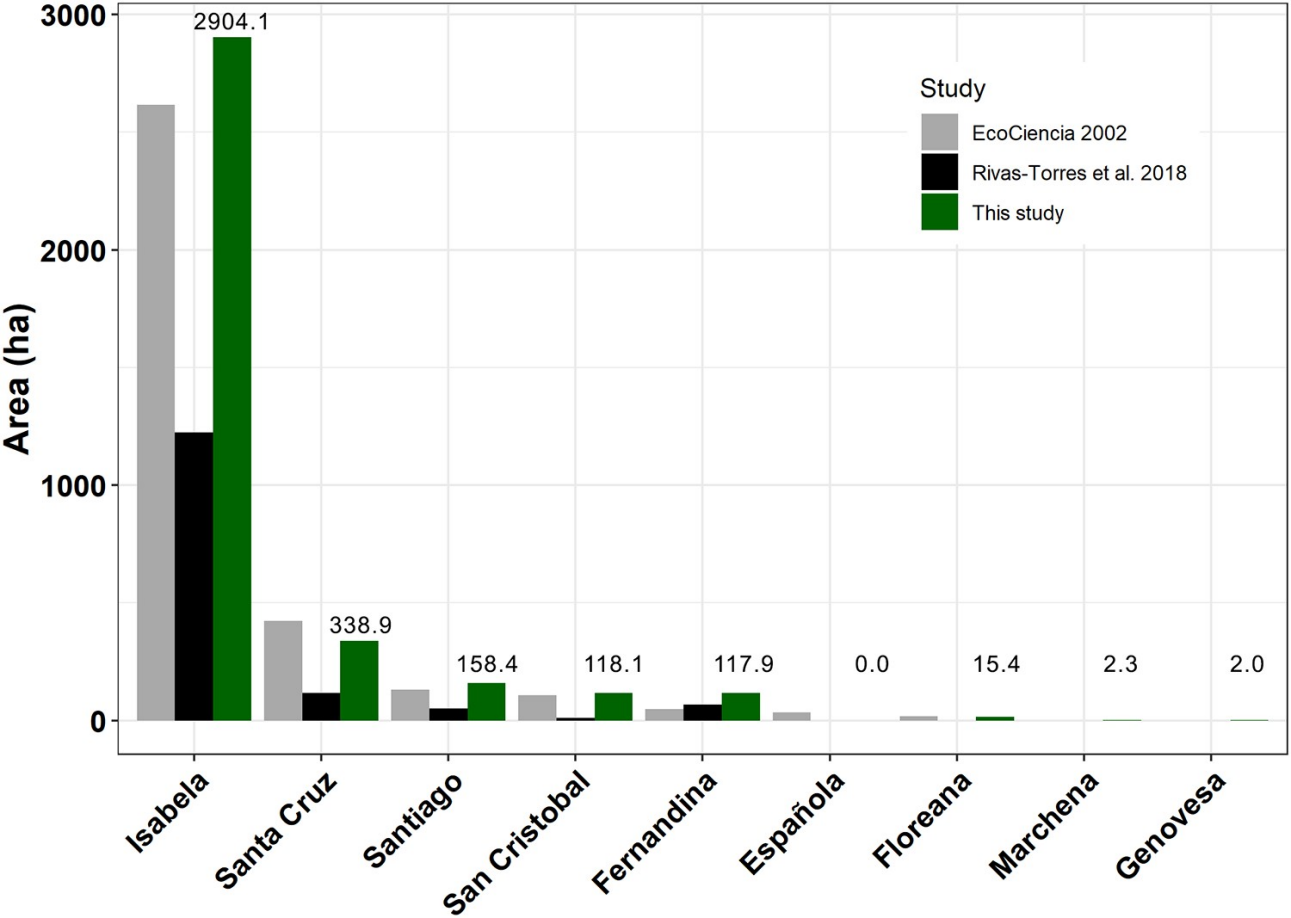
Comparison of mangrove area (ha) by island between this study, EcoCiencia and Rivas-Torres et al 2018. Numbers above the bars represent mangrove area for this study per island (ha).

**Fig 6:**
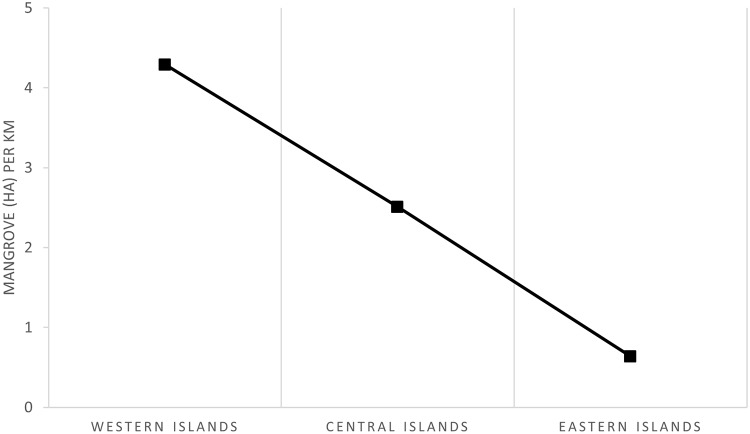
Mangrove cover (ha) per coastline length (km) by groups of islands according to their geological age.

The mangrove coverage in the Galapagos islands for year 2014 was of ~3657.1 ha, covering 35% of the coastline which benefit from the protection of mangroves (Fig 4). Spatial distribution shows that major mangrove areas are found in the island of Isabela since it is the island with most of the mangrove forests (79%) with a surface one order of magnitude higher than any other island (Fig 5). The relative area of mangrove per island (relative to the length of the coastline) shows that Isabela island has the highest mangrove density (S1 Fig). When grouping the islands per geological age, there is a trend showing that the coast of the youngest islands have more mangrove cover than the oldest ones (Fig 6).

The distribution of mangrove forests per bioregion (*sensu* Edgar et al., [79]) shows that most of the mangroves are in the central-south-eastern and western bioregions (including Elizabeth) (Fig 4); both bioregions contain ~ 90% of the area of Galapagos’ mangrove forests.

**Fig 7:**
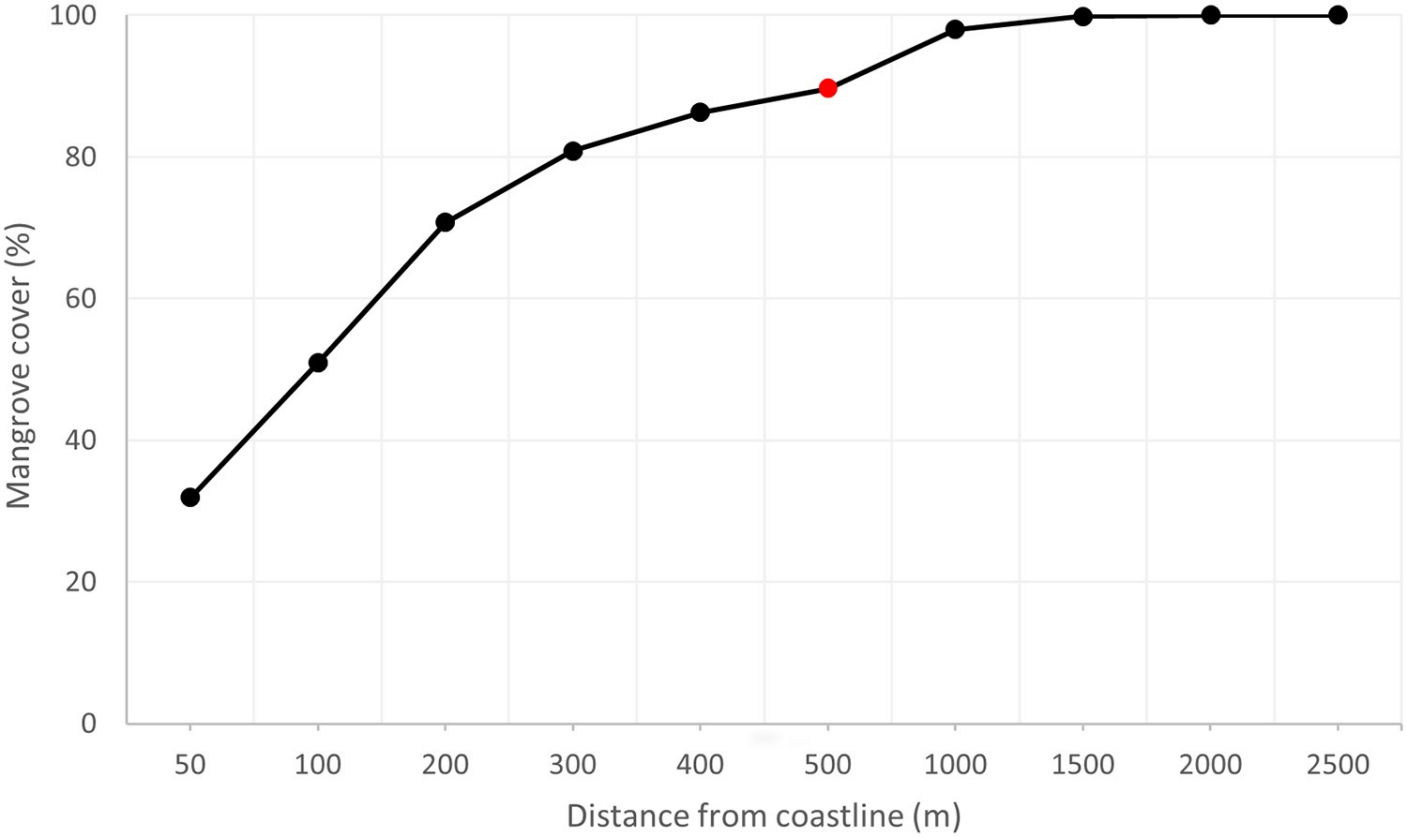
Distribution of mangrove forests with increasing distance from coastline. Red dot represents 90% mangrove cover.

Most mangrove patches of the archipelago are small, we found that ~85% of mangrove patches are <0.5 ha and 78% are <0.25 ha. Regarding the type of mangrove assemblage, an analysis of mangrove cover with distance from the coastline shows a high dependence of the mangroves to the sea water, which is common in fringe mangroves. Most mangrove forests (90% of total mangrove coverage) are found within the first 500 m from the coastline, while half of the mangrove forests are found in the first 100 m (Fig 7).

The resulting mangrove forest distribution layer has a relative scale of 1:2 500 ± 125, and is available as an ESRI shapefile and KMZ file at Pangaea, Data Publisher for Earth and Environmental Science (https://doi.pangaea.de/10.1594/PANGAEA.896684) and in S1 File.

### 3.2. Comparison with previous studies

This study found more mangrove coverage than any previous study in the Galapagos, even the Rivas et al. study [53], which was done with more recent imagery (Table 3). Our study differs in a range of 4 to up to > 50% with previous studies in the Galapagos.

**Table 3:** Mangrove area comparison between the different studies achieved in Galapagos. GMFD = Global Mangrove Forest Distribution.

Study	Year aerial/satellite image	Mangrove area (ha)	Percentage difference with this study (%)
This study	2014 (2005–2015)	3657.1	-
EcoCiencia (2002)	<2002	3379.1	4.0
INGALA (1989) [50]	1946–1985	2436.7^2^	20.0
Giri et al (2013) GMFD [54]	2000	2366.3	21.4
TNC-CLIRSEN (2006) [52]	2000	2145.9	26.0
Rivas-Torres et al (2018) [53]	2016	1470.4	42.6
Wium-Andersen (1986) [34]	1959–1960	1000.0^1^	57.1

^1^The aerial photographs did not cover all the islands of the Archipelago.

^2^The purpose was mapping the geomorphology, this value corresponds to the classes Mangrove and Low standing areas covered with mangrove and mangrove over rocks. There is information on mangrove coverage for Fernandina and Isabela only.

A comparison with the two most recent studies, Ecociencia and Rivas-Torres et al. [53] shows that EcoCiencia study overestimates mangrove coverage in Santa Cruz, Española and Floreana and underestimates in the rest of the islands, while Rivas-Torres et al. underestimates mangrove area in all of the islands (Fig 5).

A closer analysis of the differences with the previous studies can be done by means of polygon complexity analysis, which are representations of mangrove patches. If we compare the number of polygons and the average area per polygon in each study (Table 4), this study presents more polygons (~8 and ~13 times more than the EcoCiencia and Rivas-Torres et al. 2018 studies, respectively) and significantly smaller polygons than all previous studies. For all of the complexity metrics, this study reflects a higher complexity than the rest. All of the complexity measures were significantly higher (Dunn test, *p* < 0.01 and *p* < 0.05) than previous studies except for Complexity 2 between this study and Rivas-Torres et al. [53]. This reflects a much more precise alignment of polygon mangrove patches with the actual mangrove patches. This trend is shown for both overall complexity as well as for the measure of complexity for each island (see an example for the EcoCiencia study, Table 5). In addition, when taking into consideration the total number of nodes of all the polygons, this study has complexity values ~ 30 to 100 times higher than previous studies. Our polygons have a greater geographical fit with the actual mangrove patches. This translates into a better adjustment of the actual area of mangrove patches.

**Table 4:** Comparison of the number of mangrove patches, mean mangrove patch area, complexity measures and total number of nodes for all the studies on mangrove distribution estimation in the Galapagos. GMFD = Global Mangrove Forest Distribution.

Study	# Mangrove patches	Mangrove patch area (ha)	Complexity 1	Complexity 2	Complexity 3	# total nodes
This study	4250	0.9^a^	0.43^a^	40.2^a^*	156.5^a^	664 999
EcoCiencia (2002)	532	6.4^b^*	0.04^b^*	6.1^c^	42.3^b^	22 477
GMFD [54]	918	2.6^c^	0.09^c^	1.1^d^	7.3^c^	6 679
TNC-CLIRSEN (2006) [52]	103	20.8^d^	0.03^d^*	16.7^b^*	69.0^d^	7 111
Rivas-Torres et al. (2018) [53]	334	4.4^e^*	0.06^e^	11.7^ab^	41.4^b^	13 815

Different letters mean significant differences at *p* < 0.01 (Kruskall-Wallis and Dunn test).

Letters with * mean significant differences at *p* < 0.05.

**Table 5:** Comparison of the number of polygons and nodes per island between this study and the EcoCiencia study.

	# polygons		# nodes	
ISLAND	This study	EcoCiencia	This study	EcoCiencia
Isabela	1 822	246	341 040	8 173
Santa Cruz	674	170	57 013	10 498
Santiago	540	64	122 625	1 784
San Cristóbal	722	16	93 395	425
Fernandina	308	9	31 093	591
Española	0	20	0	699
Floreana	156	7	15 935	307
Marchena	14	0	1066	0
Genovesa	14	0	2832	0

### 3.3. Validation of the classification

#### 3.3.1 Digitizing and classification accuracy

The overall average error in the digitization of mangroves is estimated to be 15.6 ± 0.83%, i.e. ~85% of the digitized polygons align perfectly with the mangrove patches observed in GE VHR imagery at an eye altitude of 300 m. The island of Isabela has the least accurate digitization (Table 6). We did not find significant differences in the accuracy per digitizer (|Z| of 0.6, 1.0 and 1.5 for each of the digitizers, respectively).

**Table 6:** Result of the sampling to estimate the digitization accuracy.

Island	# Sampled Polygons	# Inaccurate Polygons	Partial Error (% ± error)
Isabela	82	37	45.12 ± 2.39
Santa Cruz	47	9	19.15 ± 1.01
Floreana	15	2	13.33 ± 0.71
San Cristóbal	77	8	10.39 ± 0.55
Fernandina	30	3	10.00 ± 0.53
Santiago	53	5	9.43 ± 0.50
Baltra	3	0	0.00 ± 0.00
Rábida	1	0	0.00 ± 0.00

We obtained an overall classification accuracy of 99.1% and a Kappa of 0.97. Our study has a consistently better overall accuracy and higher Kappa values (Table 7). The Rivas-Torres et al. 2018 study is the best of the previous studies, despite having detected a very low proportion of the mangroves of the Galapagos. It is interesting to note that besides having a lower overall accuracy, EcoCiencia and TNC-CLIRSEN have a Kappa of ~0.4 which means the likelihood of accuracy is less than 50% better than by chance alone.

**Table 7:** Overall classification accuracy and Kappa coefficient based on 500 sample points, ordered by overall accuracy. GMFD = Global Mangrove Forest Distribution.

Study	Overall Accuracy (%)	Kappa coefficient
This study	99.1	0.97
Rivas-Torres (2018) [53]	90.1	0.90
TNC-CLIRSEN (2006) [52]	88.6	0.89
EcoCiencia, 2002	77.5	0.44
GMFD [54]	89.3	0.40

The comparison between our study and all previous studies yielded |Z| values of 6.3, 6.9, 10.7 and 6.7 for Rivas-Torres et al. 2018, TNC-CLIRSEN, EcoCiencia and GMFD, respectively. This suggests that there is a statistically significant difference between our study and all previous studies at p < 0.05, which indicates that our mangrove classification study is significantly more accurate than the previous studies.

#### 3.3.2 Comparison with semi-automatic classification

**Fig 8:**
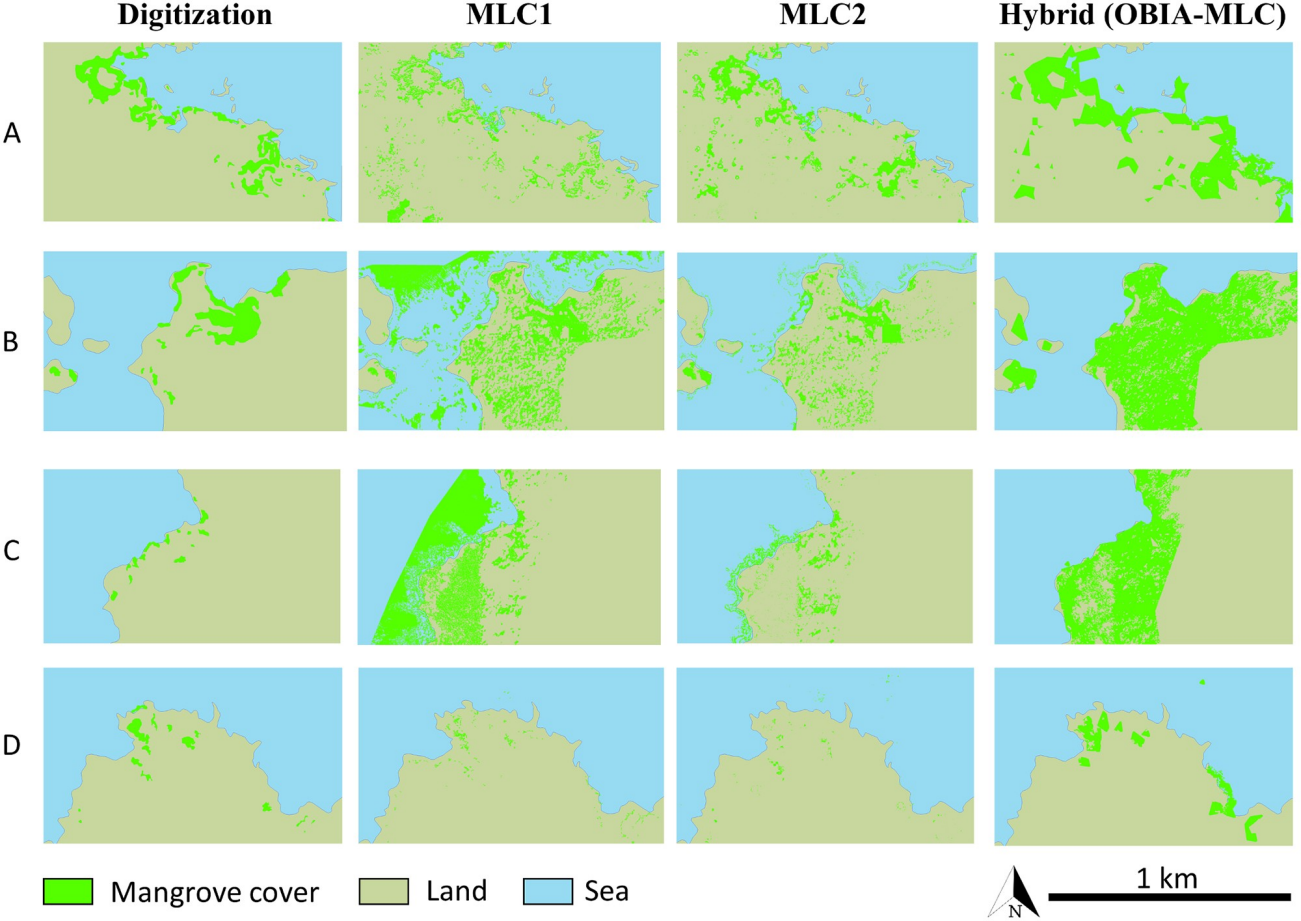
Mosaic showing the classification comparison between on-screen digitization and different Maximum Likelihood Classification (MLC) algorithms of Google Earth Very High Resolution imagery. (A) Fernandina island; (B) and (C) Santa Cruz island; and (D) San Cristóbal island. MLC1 = classification of the whole image; MLC2 = classification of the land and sea in different phases; HYBRID (OBIA-MLC) = hybrid classification technique consisting of an object based image analysis coupled with MLC. [land shapefile from the Instituto Geográfico Militar, 2013, Base Nacional escala 1:50.000].

A comparison of the accuracy between on-screen digitization and semi-automatic classification algorithms show that digitization performs better than the rest of the methods (Table 8, Fig 8). Between the semi-automatic classification methods, MLC2 is superior with an accuracy of 88.3% and a Kappa of 0.88. In all cases, on-screen digitization obtained a significantly higher accuracy (*p* < 0.05) than the semi-automatic classification algorithms (|Z| values for MLC1, MLC2 and HYBRID were of 26.5, 26.1 and 31.3, respectively).

**Table 8:** Comparison of the classification accuracy between on-screen digitization and different Maximum Likelihood Classification (MLC) algorithms of Google Earth Very High Resolution imagery. Ordered by Overall Accuracy. MLC1 = classification of the whole image; MLC2 = classification of the land and sea in different phases; HYBRID (OBIA-MLC) = hybrid classification technique consisting of an object based image analysis coupled with MLC.

Method	Overall Accuracy (%)	Kappa coefficient
On-screen digitization	99.1	0.97
MLC2	88.3	0.88
MLC1	80.7	0.81
HYBRID (OBIA with MLC)	79.7	0.80

Regarding the area classified for each semi-automatic classification method (S2 Table, S2 Fig), none of the methods give an error lower than 10% per island in mangrove estimation. From all the tested methods, MLC2 is the method that more closely matches the real mangrove area (S3 Fig). As for the islands, the youngest island (Fernandina) presented the lowest error for the three methods, while Santa Cruz was the island with more percentage error, with values over 1000% for MLC1 and HYBRID (S2 Fig). This result is probably due to the date of the images for the sampled area of Santa Cruz, which corresponded to the wet season, when the landscape turns green. In this season the tonality of the mangrove is similar to the surrounding vegetation.

When comparing the areas classified by each method, differences were only significant for the sampled areas in Fernandina (Kruskal-Wallis, chi-squared = 5.67, *p* < 0.05), and only between the HYBRID and MLC1 methods (Dunn test, Z = 2.78, p < 0.05). Despite not finding major statistical differences between the methods, the differences are very obvious (Fig 8) showing omission and commission errors due to the algorithms confounding the green non-mangrove vegetation with mangrove forests.

#### 3.3.3 Temporal change

An analysis of the mangrove cover for the three sampled islands in the 2004–2014 time span shows that there has been an overall increase of ~24% in mangrove cover which was more pronounced in Fernandina and San Cristobal than in Santa Cruz (Table 9).

**Table 9:** Mangrove cover (ha) for the three sampled areas on the islands of Fernandina, Santa Cruz and San Cristobal following on-screen Google Earth imagery digitization for images taken in 2004 and 2014. Total cover and percentage increase are also shown.

	Mangrove cover (ha)			
Year	Fernandina	Santa Cruz	San Cristobal	Total
2004	67.6	118.8	17.3	203.7
2014	95.2	130.4	26.3	251.9
Increase (%)	40.7	9.8	52.1	23.7

**Fig 9:**
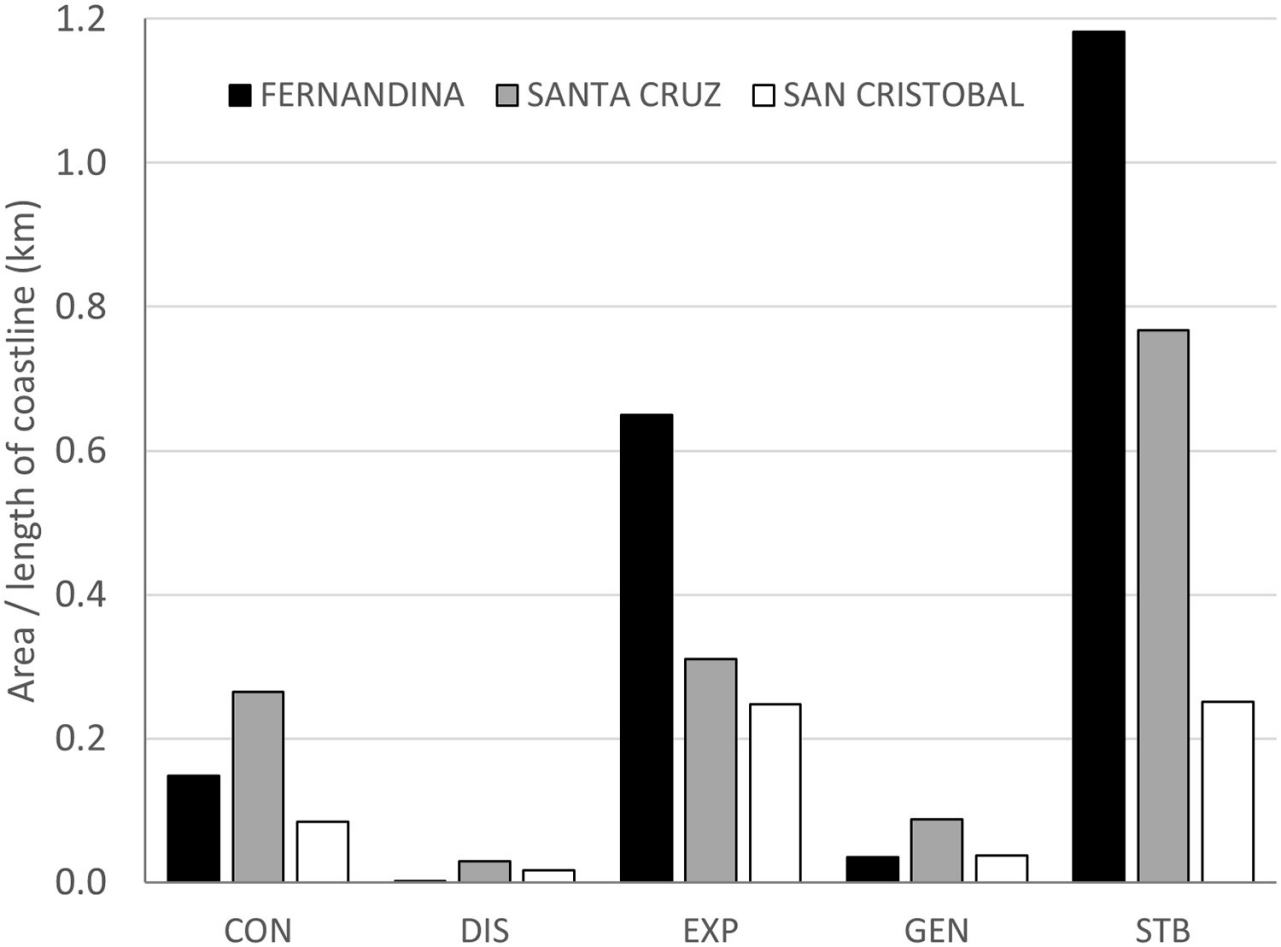
Proportion of mangrove cover change relative to the length of the coastline over the time period 2004–2014 according to a spatial–temporal analysis of moving mangrove patches for the three sampled islands. CON = contraction, DIS = disappearance, EXP = expansion, GEN = generation, STB = stable.

Comparison of mangrove patch size frequency distributions for the two time periods were only significant for Fernandina (K-S test, D = 0.27, *p* < 0.05, S4 Fig), showing that there were no big differences in mangrove patch sizes between the two time periods except for Fernandina which had a significant increase of small mangrove patches. However, a further STAMP analysis of the spatial-temporal changes of mangrove cover during the time period *t*-*t*_*+10*_, shows that the overall expansion of the mangrove cover did not occur in the same way in each island (Fig 9 and S5 Fig). As a general trend for the three sampled islands, a good proportion of the mangrove patches stayed stable, i.e. did not change their size. Also, a very small proportion disappeared, and some patches got smaller in size (contracted) mainly in the islands of Santa Cruz and Fernandina. Regarding expansion, i.e. the growth of existing patches, Fernandina is the island with greater expansion while Santa Cruz and San Cristobal had lower expansion values. The generation of new mangrove patches that did not exist in 2004 is generally low (on average 4.5%), but especially so in Fernandina.

**Fig 10:**
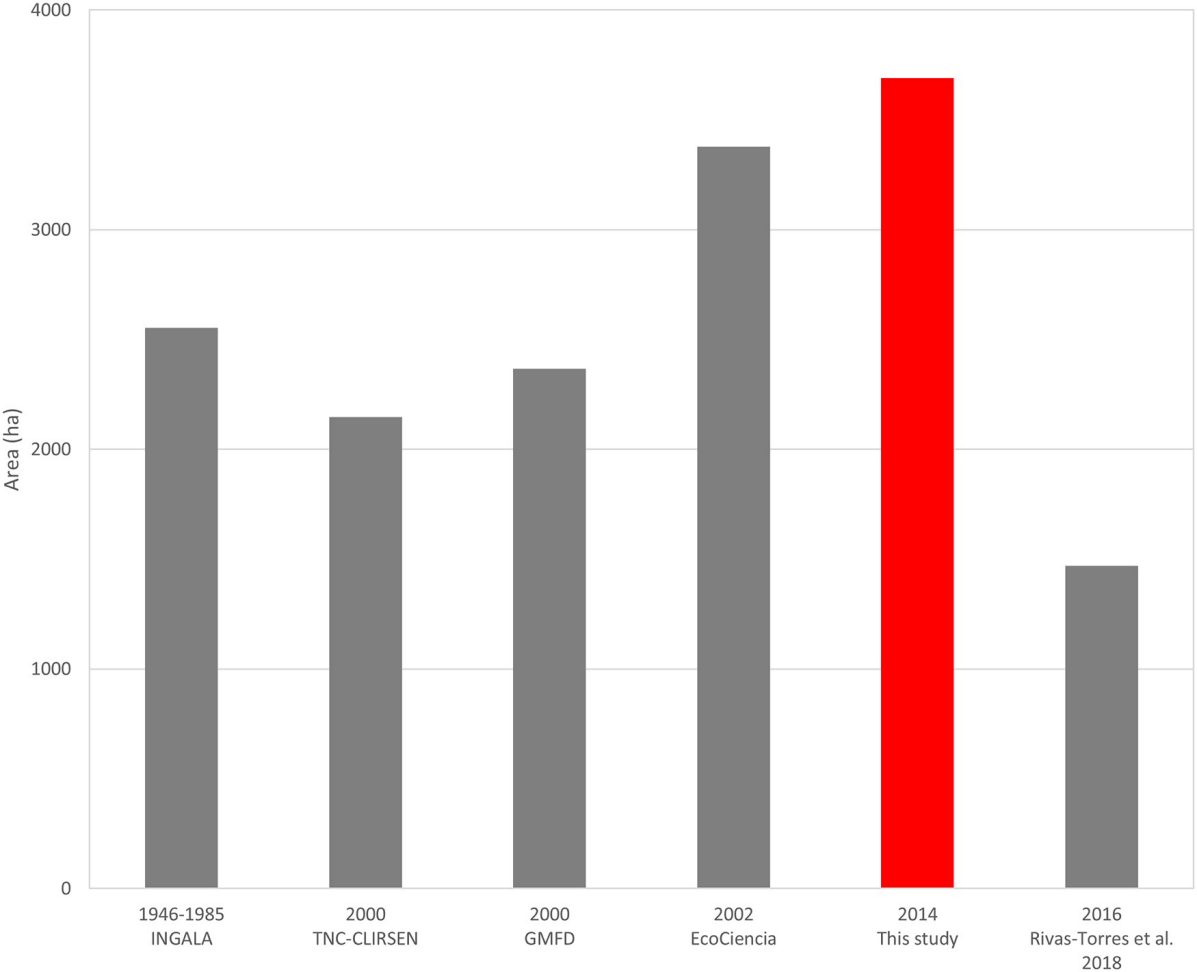
Total mangrove cover (ha) for the different studies in the Galapagos. The red bar represents this study results. The year represents the date of the most common imagery used in each of the studies.

In the Galapagos, there has been a wide range of studies covering a long time series (from 1946–1985 to 2016); however, due to differing methodologies, the results for the mangrove coverage has been different between studies and years (Fig 10).

**Fig 11:**
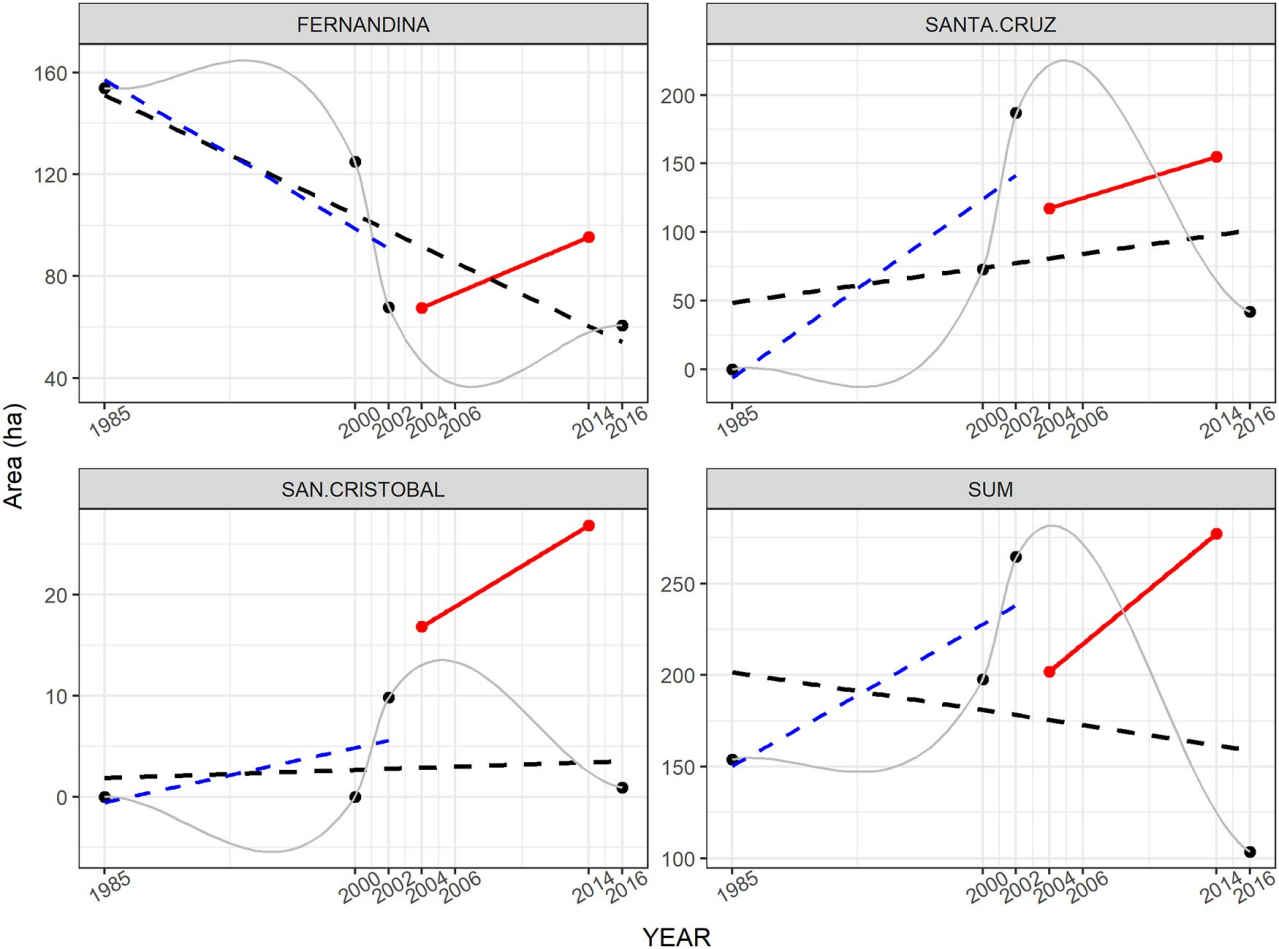
Comparison of mangrove cover for sampled areas in the islands of Fernandina, Santa Cruz and San Cristobal according to previous studies (black dots and grey line) versus our study following on-screen digitization of mangroves (red dots). Black ticked lines are linear models from previous studies, blue ticked lines are for all previous studies except Rivas et al. 2018 and red lines are for the 2004–2014 mangrove cover presented in this study. The SUM graph shows the sum of the mangrove cover of the three sampled islands. NB the vertical axis is not equivalent in all graphs. Year 2000 data is an average of the Global Mangrove Forest Distribution and TNC-CLIRSEN study, since both were done with images from 2000.

The comparison of our findings regarding mangrove cover increase in ten years with the tendencies shown by previous studies for the same sampled areas show that mangrove cover has been fluctuating with peak coverage in 2002, except for Fernandina which had its maximum coverage in 1985 (Fig 11). The general tendency (linear regression) has been an overall decrease of the mangrove cover in the 1985–2016 time frame. However, our results for the 2004–2014 change, show the opposite, an increase in mangrove cover in all the sampled areas. A further analysis eliminating the Rivas-Torres et al. study [53], which has very low values of mangrove cover, showed that the general tendency for the previous studies’ time-series is to the overall increase in mangrove cover, the same tendency we found in the *t*-*t*_*+10*_ mangrove cover analysis (Fig 11).

## 4. Discussion

### 4.1. Mangrove forest distribution and dynamics in the Galapagos

This study demonstrates that the Galapagos mangrove forest is not distributed equally among islands. There is a clear west-east mangrove coverage pattern in which mangrove cover of the coastal zone is inversely proportional to the geological age of the islands, i.e. geologically young islands have more mangrove cover than older islands. Interestingly, the west of Isabela has a relative mangrove cover of 3.26 ha/km while the east of 3.20 ha/km and Fernandina of 0.69 ha/km. This shows that the distribution of mangroves is not equally distributed spatially within the youngest islands. There is also a clear relationship between geological age and lava cover in the coastal area, younger islands have a higher proportion of lava cover while older islands of deciduous vegetation. Thus, mangrove forest could be interpreted as pioneering vegetation, as it has already been stated in other studies [34,83] and not very competitive with non-mangrove vegetation when soil meteorization has taken place. However, most probably this finding is the result of a series of factors in addition to geological age of the island and lava cover, such as water nutrient content, wave exposure protection and current dynamics as will be explained hereafter.

Our temporal mangrove cover analysis from 2004 to 2014 shows an overall increase of the mangrove area. However, the tendency from previous studies show the opposite, there is a general decrease of mangrove cover in the Galapagos since 1985. We demonstrated the strong effect that the 2016 [53] study has on this tendency. By eliminating the values from that study, the linear regression shows an overall increase in mangrove cover much more similar to what we found for most of the islands. These results show that the method used by Rivas-Torres et al. [53], while providing a global vegetation cover for the entire Galapagos archipelago, is probably better targeted at vegetation detection in the humid zone than in the coastal zone. Also, it shows the great discrepancy between previous studies, for example, there are three studies for year ~2000 that show a total mangrove area ranging from 2100 to 3400 ha, this is most probably due to differing methodologies. Our results, with a global percentage increase of ~24% in mangrove cover in ten years (2.4% annual growth) are in par with other studies analysing mangrove cover temporal changes. A study in Malaysia showed an annual increase of 3.2% for a six-year study, while in Vietnam, an annual increase of 1.3% was found for a 20-year study [72,84]. Yet, the results from these studies show an overall increase of the gain-loss equation, since these areas suffered from serious human intervention both in mangrove loss and in mangrove reforestation. The Galapagos mangrove forest is virtually free of human intervention, practically no loss can be attributed to human causes (except in inhabited islands which were not selected in the sampled sites) and there is currently no reforestation program for mangroves. The annual growth value is probably lower than in other tropical areas if they were left as primary forest, because Galapagos is not ideal for mangrove development since there are no permanent freshwater sources nor estuaries.

These results show the importance of using the same image classification method to evaluate mangrove growth dynamics in order to get reliable results. In the case of arid tropical areas, such as the Galapagos coastline, mangroves are small and changes may remain undetected by other methods based on coarse image resolution. Here, we showed that most mangrove patches are small (<0.5 ha), however, the temporal changes are noticeable and the growth rate comparable with other tropical areas. The way mangroves grow appear to be mainly by patch expansion rather than by the creation of new patches, i.e. existing patches get wider and colonize new areas. These findings are interesting and would benefit from being confirmed with on-site monitoring to validate the trend and understand the ecological processes behind them. Although there are many studies on the temporal change of mangroves, they focus on mangrove accretion and erosion at wider scales but not much on spatial–temporal analysis of moving polygons. To our knowledge, this is the first time this method has been used for mangrove growth dynamics.

The differences in mangrove cover and growth rate between islands may be due to multiple reasons. The west of the archipelago is known for having cold waters rich in nutrients, associated to the Cromwell current upwelling (also called Ecuadorian under current, EUC), while the central islands get the influence of the warm Panama current coming from the northeast and the cold Humboldt current coming from the southeast [85,86]. Hence, general mangrove cover and growth in the west should be impeded by SST since it is significantly colder than in the rest of the archipelago [86]. However, our temporal change analysis show that the western bioregion has the highest overall mangrove increase in the ten-year span, and highest relative mangrove cover (2.65 ha/km versus 1.55 ha/km for the central-south-eastern bioregion). It is the area with most stable mangrove patches, more expansion, very low contraction and almost no disappearance. This seems to be in accordance with other studies demonstrating that SST may not completely limit mangrove distribution for two of the genera also found in Galapagos, *Avicennia* and *Rhizophora* [87]. Nutrient content might be another explanation for the wellness of Fernandina’s mangroves and the general mangrove cover of the western bioregion. The west of Isabela-Fernandina area is the most influenced by the EUC upwelling [86] and has nutrient rich waters, which seem to favour mangrove growth [88]. However, salinity seems to be higher than in other parts of the archipelago not influenced by the upwelling, 34.5–35 PSU, [89], which might handicap mangroves [90] since there are no estuaries nor permanent rivers supplying fresh water in the Galapagos coastline. Yet more recent measurements show a difference of only 1 PSU [91] or < 0.5 PSU [92] between the west of Isabela and the central-south-eastern bioregion, so probably salinity is not influencing much mangrove distribution. In addition, studies support that mangroves are facultative halophytes [93] and that salinity provides a competitive advantage by exclusion of non-mangrove vegetation rather than being a physiological need [90], so the influence of salinity in mangrove distribution is complex and need site specific measurements. In addition, the sampled area of Fernandina is a channel, well protected from wave exposure. Geological age, wave protection and nutrients seem to be increasing mangrove growth, stability and, ultimately, cover, in this area and may be an explanation for the differential mangrove cover between the western and central-south-eastern bioregions. Also supporting the idea of a multiple factor explanation is the fact that Fernandina, despite being completely influenced by the EUC nutrient rich waters, has mangroves growing only in the eastern coast, facing the channel and protected from wave exposure, while being completely absent on the west side of the island which is exposed to waves. This suggests that nutrient alone cannot explain the differential mangrove distribution pattern. Interestingly, the eastern side of Fernandina island was identified as one with highest impact for the 2011 Tsunami [94]. However, its mangrove forest does not seem to have suffered much, although a detailed analysis of the change prior and after the Tsunami would be needed to uncover if there was an impact. The highest contraction value found at Santa Cruz might also be due to the Tsunami since the north of the island was also identified as an impacted site. Most probably, the lower expansion and stability rates found in Santa Cruz and San Cristobal islands, reflect the influence of the age of the island on its adequacy for mangrove development. It is interesting to note that Santa Cruz and San Cristobal have more new mangrove patches than Fernandina, this might be due to mangrove propagule transportation towards and within the islands. This requires further research in genetic connectivity within and between islands, and with other places outside of the GMR since current patterns are very complex in the Galapagos [89].

The idea behind this temporal analysis was mainly to test if it was possible with all the previous studies, and showed the importance of using the same methodology for temporal change analysis. However, it is a very interesting field of research since most of the mangrove dynamics studies show a general tendency of decreasing mangrove cover in the tropical belt due to multiple human or human-induced stressors (see for example [27,95]). Thankfully, our data shows the opposite, which may be a result of the protection of this archipelago since 1959. Mangrove forests in the Galapagos are close to a pristine-state and virtually free of major human impacts.

### 4.2. Mangrove cover mapping

The high complexity of digitized polygons, the accuracy and finer scale of the digitized polygons and the high overall accuracy highlight the reliability of our methodology and demonstrate that our updated maps of mangrove coverage are more reliable than previous maps created with supervised and unsupervised classification of satellite imagery.

Our study is the first to attempt the use GE to map mangrove coverage in the Galapagos and has resulted in the most precise mapping achieved so far with an overall accuracy and Kappa estimates significantly superior to the GMFD, EcoCiencia and Rivas-Torres et al.’ [53] estimates. Indeed, the GMFD has its own errors because they conducted a global mangrove mapping at a cell size of 30 m and the methodology does not allow for the identification of mangrove patches smaller than 900–2700 m^2^ [54]. Similarly, Rivas-Torres et al. [53] also uses Landsat imagery with a minimum mapping unit of 2500 m^2^. Yet, our study demonstrates that most (~80%) of the patches in the Galapagos are of less than 2500 m^2^, so virtually undetectable by the GMFD and Rivas-Torres et al.’ [53] studies. Hence, the image classification methodology used in these studies proves not being reliable to detect mangroves in the Galapagos area. Indeed, despite using 15 m pixel resolution, the highest of all previous methods, we have detected one order of magnitude more mangrove patches than the Rivas-Torres et al. [53] study. Similarly, the EcoCiencia study relies on Landsat imagery analysis and has similar problems detecting small mangrove patches. The two most recent studies, EcoCiencia and Rivas-Torres et al. [53], were not aimed at mapping only mangrove forests in the Galapagos but rather the vegetation cover of the islands. This explains why we achieved better results, especially in the case of Rivas-Torres et al. [53], that underestimated mangrove area by at least 40%.

Although satellite remote sensing offers many advantages to gather mangrove information, they also have drawbacks. Some drivers of change are not detectable at the scales offered by freely available imagery data (e.g. Landsat, Sentinel) and potentially require other sources of information [19]. Small, isolated patches of mangroves are usually totally overlooked by medium-resolution platforms, which have a pixel size in the range of 30 to 10 m, but are detected by high resolution optical imagery [25,96,97]. Yet these small patches, although making relatively little difference to the global mangrove area of an area or region (8% in the case of Galapagos for <0.5 ha patches), can be key in ecological processes, as it has been demonstrated for coastal fisheries in the Gulf of California [98]. Here, we demonstrated that Galapagos’ mangroves are fringing mangroves, occupying the first 500 m of coastline. Mackenzie et al. [19] showed in their study that most of the mangroves in a Vietnam province also occupied a thin fringe of the coastline and were highly fragmented and difficult to detect using Landsat imagery. Fringing shoreline mangroves, such as the Galapagos mangroves, are highly relevant for the provision of ecosystem services with their contribution to coastal productivity [19,98,99], as well as sediment and nutrient retention, and wave energy attenuation [9,100]. The importance of high spatial resolution mapping of mangroves and the utility and power of mapping from visual interpretation and on-screen digitization has already been highlighted by others [27,28,101]. The high resolution is particularly useful for ecological studies as it is the resolution at which ecological processes occur.

While on-screen digitization of GE VHR imagery is tedious and time consuming, other techniques like automatic or semi-automatic image classification of satellite imagery require a level of technical expertise of personnel and technological needs usually not available at the management level in tropical regions where mangroves occur [102]. Other methods, like visible vegetation index discrimination or NDVI prior to MLC did not yield better results. OBIA coupled with MLC did not give good results either, however it is usually identified as the best method in other satellite imagery classification studies since it can incorporate co-variables such as distance to the coast, elevation, currents or wave energy as it has been done in other marine ecosystems [103]. As Collin et al. found [71], MLC with prior segmentation of the image for terrestrial and marine habitats yielded the best results from the semi-automatic classification methods for classifying RGB images. Even though others had good results with semi-automatic classification of RGB imagery [71], we obtained better results with on-screen classification and do not recommend using semi-automatic classification of GE RGB images to detect mangroves. Without any further information than the red, green and blue channels it is very difficult for the algorithm to discriminate between the digital numbers that compose mangrove patches and other vegetation or even water surfaces with algae. Prior differentiation of the vegetation with NDVI from Sentinel imagery did not yield better results either.

**Fig 12:**
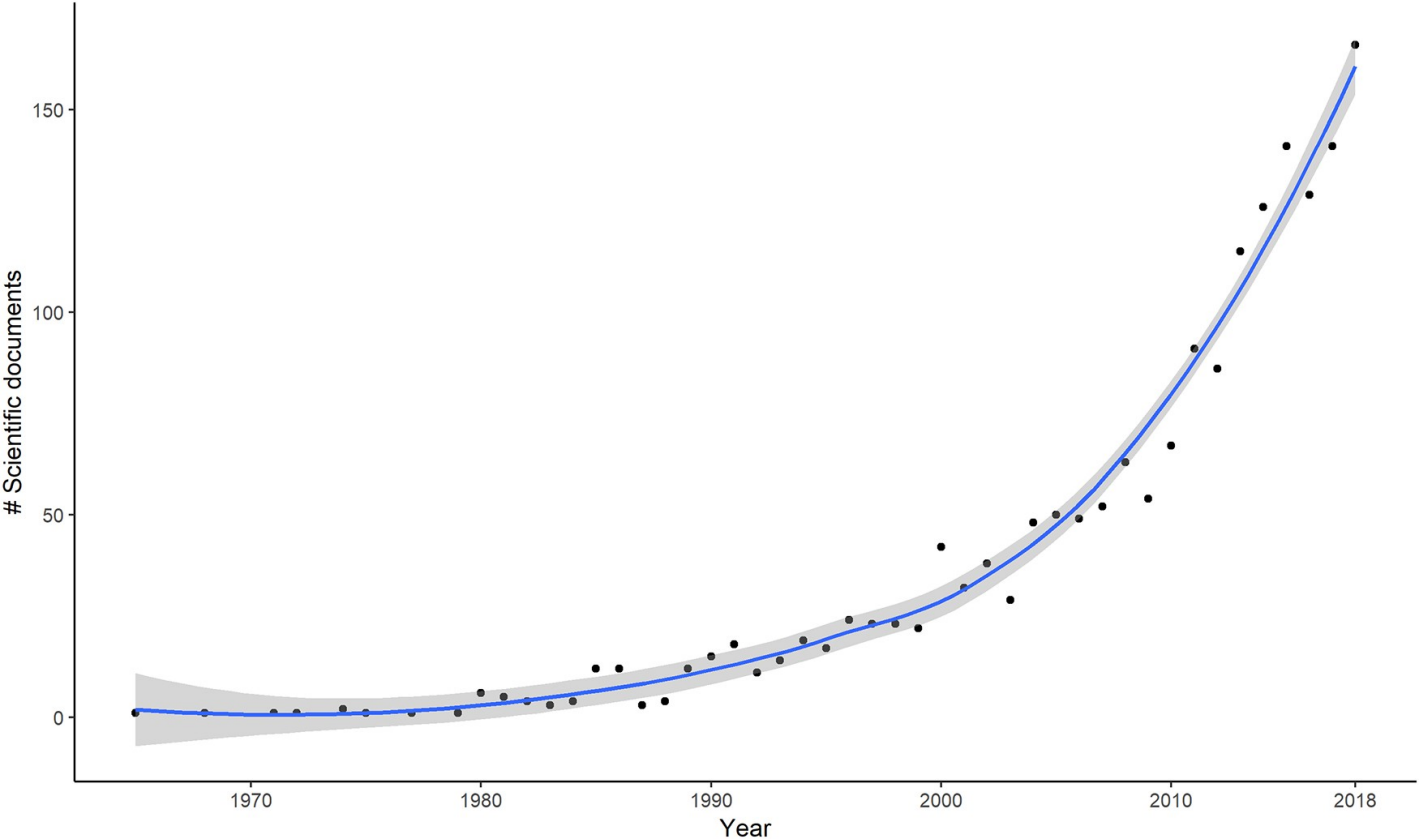
Publications on mangrove mapping or mangrove cover from 1965 to 2017 in the Scopus indexed database.

Although the mapping of mangrove forests has received much attention from the global scientific community since the 2000s (Fig 12), GE has rarely been used as a main source to detect the presence of mangroves.

Many mangrove mapping studies have used GE VHR to validate the presence of mangroves, e.g. in Galapagos [53], SE Asia [95], USA [104], Papua [23], Sulawesi [24], or globally [55]. GE has been used to map land cover [69,105], to delineate ecosystems [106], map rock glaciers [107], and identify atolls [108] or coastal engineering structures [109]. However, few studies attempted to use GE to map coastal or marine ecosystems. Hossain et al. [110] used GE to map seaweed culture sites, but relied on Landsat to map the rest of the marine and human habitats. Aburto-Oropeza et al. [98] digitized isolated, small patchy mangroves directly in GE, after *in situ* ground-truthing the mangrove pockets to complement more coarse Landsat mapping. Collin et al. [71] used GE to map seabed and land cover with MLC. They downloaded the GE images to create a mosaicked composite of the study area to perform a Maximum Likelihood semi-automatic supervised classification whereas we identified the mangroves visually and drew the area manually, within GE software. While our visual method might not be appropriate in non-volcanic islands, where mangrove identification could prove more difficult, Collin’s method presents a problem for mapping large areas, like the Galapagos coastline, because GE images need to be downloaded for local analysis. A rough estimate of the number of images needed to cover the Galapagos coastline (2058 km) using this method is ~6700 images which would need to be selected, downloaded, corrected and analysed. Our research demonstrates that the time invested to download the images, process and analyse them with the appropriate algorithm, does not always compensate for the quality of the end product. Indeed, the three methods that we tested, using a semi-automated classification of GE Red Green Blue (RGB) imagery were consistently less accurate than on-screen digitization and missed a high percentage of mangrove cover.

The high overall accuracy and kappa coefficient (99.1% and 0.97, respectively) that surpasses other mangrove mapping studies [23,71,95] points to the reliability of our approach. It demonstrates that using GE VHR imagery as a substitute to aerial imagery is useful for high resolution mangrove mapping, as Aburto-Oropeza et al. [98] and Collin et al. [71] showed in their studies. Furthermore, the VHR nature of GE imagery allows for the detection of small pockets of mangrove, which in tropical arid environments can represent an important proportion of the total mangrove forest [98] or even in other more humid tropical areas, such as Vietnam [19].

The accuracy comparison per digitizer shows that having several individuals digitize the polygons does not compromise the accuracy as long as a good visual interpretation attribute table or key is thoroughly made before the digitization. These have shown to be fundamental in other visual interpretation studies, however, the limitation of such interpretation keys lies in its inability to apply it to other mangrove regions of the world with different species, compositions, and environmental conditions [101].

As it has been found in other studies, our findings support that mangrove mapping is most effective when the mangrove patches lie as independent features surrounded by water, lava or vegetation-free areas on the inland side of the mangrove assemblage [22,111]. These land-cover changes are more easily detected by classification algorithms and by human interpretation. In this study, we found that it was easier to detect mangroves on the younger islands, which are characterized by independent mangrove patches surrounded by lava or water, than in older islands, where deciduous vegetation meets mangrove forests. It is thus very important not to rely entirely on the classification algorithm but to include, where possible, sources of mangrove verification like previous mangrove classifications, field studies and, if it is available, expert and traditional ecological knowledge.

The complexity measures that we propose, are, by no means determinants of mangrove presence. However, they represent an interesting measure of polygon complexity which, if coupled with the rest of measures (like accuracy and an image interpretation attribute table), can be another indicator of the quality of a mangrove classification method. Accuracy alone does not guarantee a high detection of mangrove presence. For example, the Rivas-Torres et al. study has a very high accuracy (90%) but is missing ~40% of mangrove cover in the Galapagos.

### 4.3. GE VHR as a cost-effective solution

Our results support the idea that GE VHR imagery can be used to effectively map mangrove forests in tropical and subtropical arid and semi-arid environments. This showed to be true for on-screen digitization, where mangrove areas in an arid climate will be more easily discriminated from other vegetated areas, as well as for Maximum Likelihood Classification algorithms. Since GE VHR imagery is freely available, this imagery source proves particularly valuable for mapping natural resources in developing countries and marine protected areas with constrained financial resources. A cost-benefit analysis of the various approaches shows that the GE solution offers a much more efficient means for achieving the same results (between 400 and 35 000% cheaper) (Table 10). The Digital Globe Satellite Imagery has the advantage of being multispectral, but the analysis of this kind of imagery may require the acquisition of expensive proprietary software, namely remote sensing or object-based analysis software (like e-Cognition). Mapping with unmanned aerial vehicle (drone) images would be much more precise, but the price and carbon footprint to get the images would be higher due to the need for logistical support from a vessel to access the entire coastline of the islands. New advances in photogrammetry from manned-aircraft are promising as they can yield centimetre to decimetre resolutions at a relative low cost [112]. However, the mangrove area to be mapped in the Galapagos Archipelago is huge (~4000 km^2^), and the flight hours considerably increase the cost.

GE VHR imagery can thus supply the scientific community with reliable information on spatio-temporal changes of ecosystems and the anthropogenic impacts, the distribution of ecosystems and effectiveness of management measures and policy [102].

**Table 10:** Cost-benefit of different solutions to map Galapagos’ mangroves. Analysis and image processing involves the working hours of technical staff and/or software. MMU = Minimum Mapping Unit, RGB = Red, Green, Blue; USD = United States Dollar.

Solution	Scale	Images Price (USD)	Image Spectral properties	Analysis and image processing (USD)	Total (USD)
Google Earth Pro	1:2500 (estimated, MMU 10 m^2^)	0	RGB	15 450	15 450
Digital Globe Satellite Imagery	1:6000	65 900	Multispectral	15 600^1^ 20 100^2^	81 500 86 000
Photogrammetry from manned-aircraft	1:200–1:20	150 000	RGB / Multispectral	15 600	165 600
Unmanned aerial vehicle (drone)	1:2000	5 429 474	RGB	15 450	5 444 924

^1^ machine learning image analysis using R Statistical Computing;

^2^ object-based image analysis using e-Cognition software.

We developed a cost effective methodology using free GE images and GIS programs which enables countries and institutions with fewer economic resources to conduct extensive surveys and sound analyses of natural resources needed to inform resource management efforts.

### 4.4. Limitations

As it has been pointed out by other authors [69,105–107,113], using GE imagery for research has some limitations that need to be addressed to make the best use of this valuable tool. Positional accuracies in GE vary in different parts of the world [81] and should be assessed on a region by region basis. Furthermore, not all tropical regions of the world have high-resolution images. The acquisition dates of the images are inconsistent, making large area analysis a challenge, especially with rapid changing ecosystems. GE images are further limited spectrally to a three-band colour code (RGB) because they are intended for visual purposes, not remote sensing analysis. Hence, analysis on multispectral combinations are limited to RGB, reducing the range of analysis and classification procedures, as we showed with the poor results of MLC and RGB vegetation indices. In addition there are inconsistencies in radiometric distortion and the metadata of the provided images is still largely unknown [113]. Therefore, GE imagery should be used with caution.

Positional horizontal accuracy in GE imagery, or the accuracy of the mapped object in relation to the true position of the object, is one of the greatest concerns with using GE in science and there still exists many uncertainties regarding this topic. Horizontal accuracies have been estimated from 113 m of misalignment [114] to 40 m around cities [115] or even down to 1m in Rome [116]. In rural areas of the world misalignments range from 10.5 m [81,117] to 4.1 m [118]. Since the horizontal positional accuracy of GE is very much dependent on imagery resolution [118], a fairly good estimate of the positional accuracy is derived from the resolution of the imagery used in our study, which was very high for the Galapagos coastline. Thus, we estimate the positional horizontal accuracy to be sufficient for the mapping of mangrove forests in the Galapagos. Regarding the errors in measured length, width and surface area of human and natural features from GE derived polygons, it has been proven that GE measurements and on-the-ground measurements are in agreement (R^2^ = 0.982) along 350 km of the Australian NE coastline [109], hence we are confident in the measurements calculated in this study.

In very densely vegetated coastal areas of the world which do not allow an easy differentiation of mangrove forests from other vegetation (with similar texture and tone), on-screen or semi-automated classification methods with GE VHR RGB images may be of limited use. In those cases, a first level of discrimination should be done with multispectral imagery and with the help of vegetation indices, such as NDVI, although this might not solve the classification difficulties, as we showed here.

One way to get higher digitizing precision would be to use very high resolution imagery free of clouds with a high spectral and temporal resolution. This would enable an object-based image classification analysis combined with algorithms of supervised learning [119]. Such methodology would pave the way for the automated differentiation of mangrove patches from other coastal vegetation, such as the salty bush (*Cryptocarpus pyriformis*), a very common species found along the Galapagos coastline quite easily confounded with mangrove forests in RGB images. Furthermore, it could potentially lead to mangrove species differentiation. However, it has already been demonstrated that, in the Galapagos, the specific composition of the mangrove patches cannot be differentiated with this technique [120].

Although we based our overall classification on numerous field trips, we recommend ground truthing more areas. This would allow researchers to critically evaluate the tested classification methods and get an even better estimate of the accuracy across the archipelago. The best approach to mangrove monitoring and mapping is probably having a multidisciplinary integrated GIS approach which combines remote-sensing and on-ground reference data with the input from expert-knowledge, when available [19,101].

### 4.5. Conclusion

In this study we successfully used GE VHR imagery to accurately map mangrove forests in the Galapagos and recommend this imagery source as a valid method for mapping mangroves, especially in tropical and subtropical arid and semi-arid areas. The study highlights the importance of freely available data sources to the public domain which can be used to aid in decision making regarding biodiversity conservation and spatial planning. Freely available very high resolution satellite imagery of the entire Earth surface is now available thanks to GE and it allows the study of ecosystem and landscape features in the most remote areas, including difficult to access habitats. This opens an all-new era of Earth observation and study to even developing nations or organisations. Our results add to the growing body of literature that supports the use of GE as a source of imagery to map natural features.

The mangrove map presented in this study is a fundamental layer that will be useful for a plethora of research areas such as fisheries, carbon sequestration, ecosystem services identification and valuation, Blue carbon estimation, biodiversity, conservation, restoration, tourism, coastal protection, economic evaluations, spatial planning, governance and climate change. The mapping of mangroves is also essential to produce an accurate baseline for time-series change analyses, as well as to facilitate research on the dynamics of mangrove communities and their relationships with biophysical factors, and to understand responses to different drivers. Ultimately, our mangrove forest map represents an important updated reference to support the conservation and management of mangrove forest resources along the Galapagos coastline. It is now available to natural resource managers as a decision-making tool for sustainable management of mangrove ecosystems in the Galapagos.

## Supporting information

S1 Fig Mangrove cover (ha) per length of coastline per island. (DOCX)

S2 Fig Percentage error of mangrove classification for each of the semi-supervised classification methods, averaged per island. (DOCX)

S3 Fig Distribution of mangrove patches area per classification method. (DOCX)

S4 Fig Mangrove patch size (ha) frequency distribution per island and year. Vertical lines represent mean of mangrove patch size for each year.
(DOCX)

S5 Fig Spatial–temporal analysis of moving mangrove patches for the time period 2004–2014 for the three sampled islands (A = Fernandina, B = Santa Cruz, C = San Cristóbal). CONT = contraction, DISA = disappearance, EXPN = expansion, GENR = generation, STBL = stable. Background screen captured from World Imagery Esri Tile Layer. Locate in: https://services.arcgisonline.com/ArcGIS/rest/services/World_Imagery/MapServer.
(DOCX)

S1 Table Google Earth image date and location within the Galapagos islands used to digitize mangroves in the Galapagos. (DOCX)

S2 Table Mangrove cover (ha) per classification method, island and sampled section. MLC1 = classification of the whole image; MLC2 = classification of the land and sea in different phases; HYBRID (OBIA-MLC) = hybrid classification technique consisting of an object based image analysis coupled with MLC.
(DOCX)

S1 File ZIP file of Galapagos mangrove distribution (ESRI Shapefile and KMZ files). (ZIP)
