# Correction: Mangroves in the Galapagos islands: Distribution and dynamics

**DOI:** 10.1371/journal.pone.0212440

**Published:** 2019-02-11

**Authors:** Nicolas Moity, Byron Delgado, Pelayo Salinas-de-León

## Notice of republication

Incorrect versions of Figs 8 and S1–S5 were published in error. This article was republished on February 1, 2019 to correct for this error. Please download this article again to view the correct version.

## References

[1] Moity N, Delgado B, Salinas-de-León P (2019) Mangroves in the Galapagos islands: Distribution and dynamics. PLoS ONE 14(1): e0209313 10.1371/journal.pone.0209313 30625180 PMC6326481

