# Supplementary figures and images for: Mangroves in the Galapagos islands: Distribution and dynamics
Source: PLoS One. 2019 Jan 9;14(1):e0209313. doi: 10.1371/journal.pone.0209313 (PMC6326481; doi:10.1371/journal.pone.0209313)

**S1 Fig. Mangrove cover (ha) per length of coastline per island.**


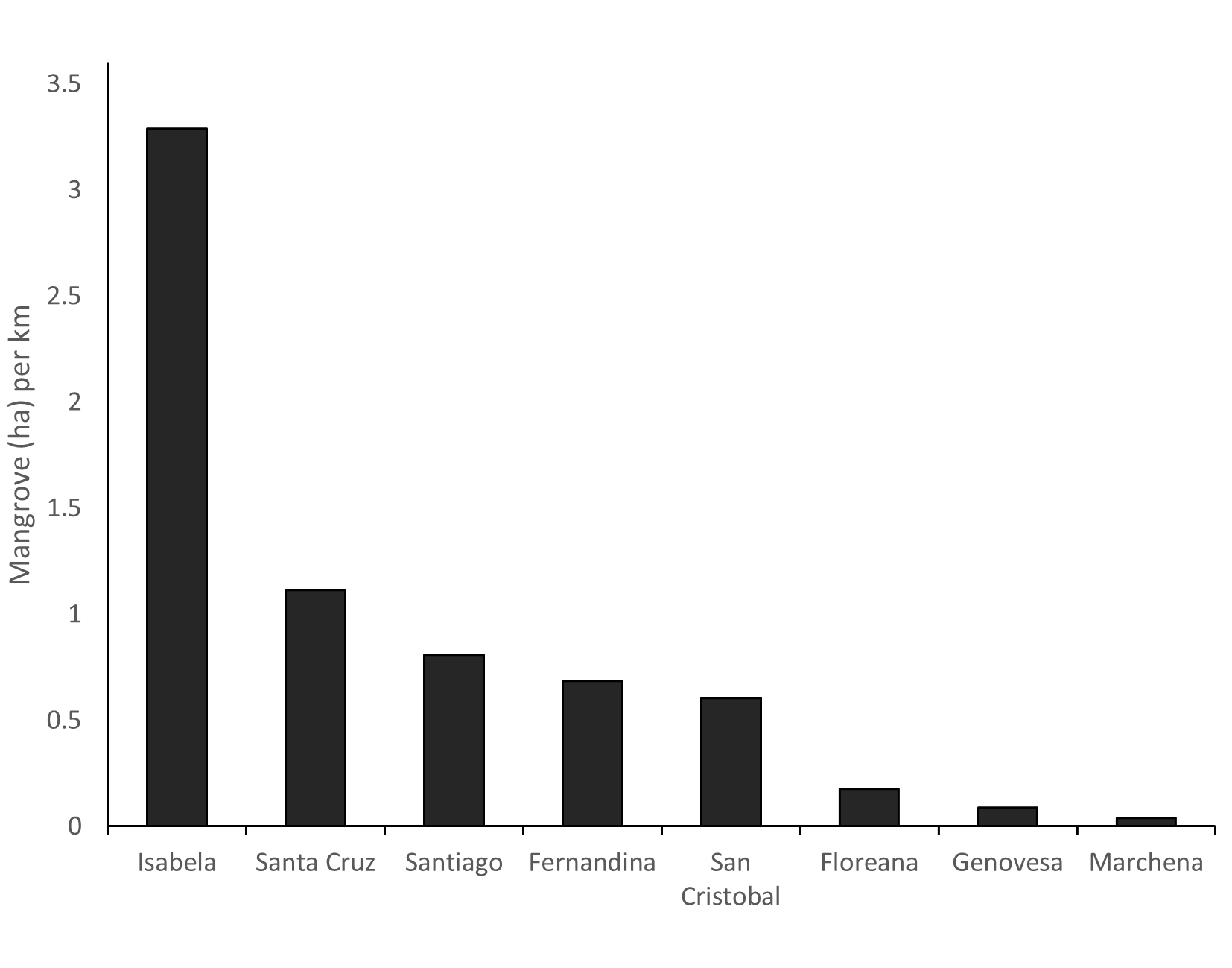

Supplement: S1 Fig — (DOCX) [file pone.0209313.s001.docx]
