# Supplementary material for: Mangroves in the Galapagos islands: Distribution and dynamics
Source: PLoS One. 2019 Jan 9;14(1):e0209313. doi: 10.1371/journal.pone.0209313 (PMC6326481; doi:10.1371/journal.pone.0209313)

**S2 Fig. Percentage error of mangrove classification for each of the semi-supervised classification methods, averaged per island. MLC1 = classification of the whole image; MLC2 = classification of the land and sea in different phases; HYBRID = hybrid classification technique consisting of an object based image analysis coupled with MLC.**


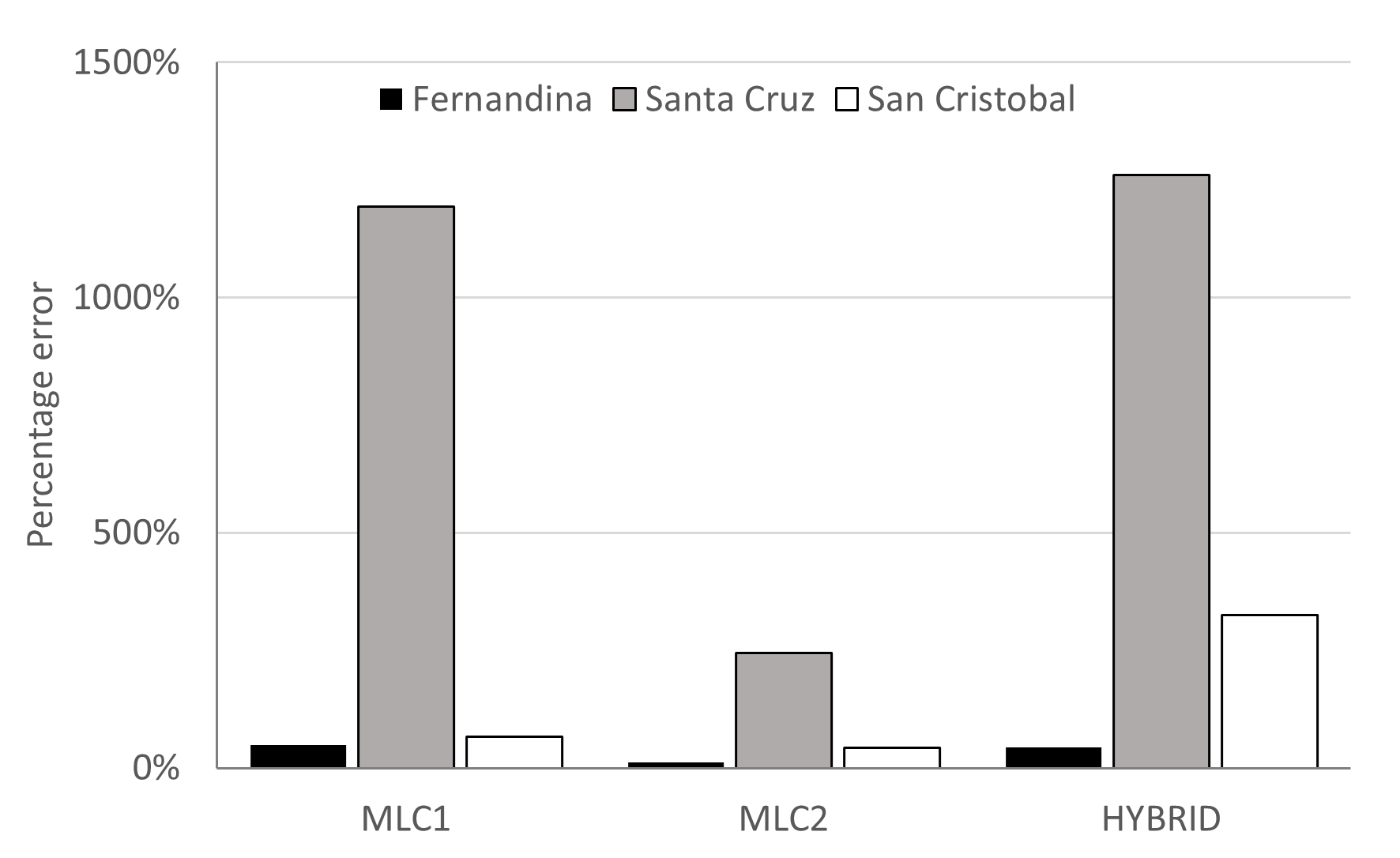

Supplement: S2 Fig — (DOCX) [file pone.0209313.s002.docx]
