# Supplementary material for: Mangroves in the Galapagos islands: Distribution and dynamics
Source: PLoS One. 2019 Jan 9;14(1):e0209313. doi: 10.1371/journal.pone.0209313 (PMC6326481; doi:10.1371/journal.pone.0209313)

**S3 Fig. Distribution of mangrove patches area per method. Points represent the raw data and the bean represents the density of points.**


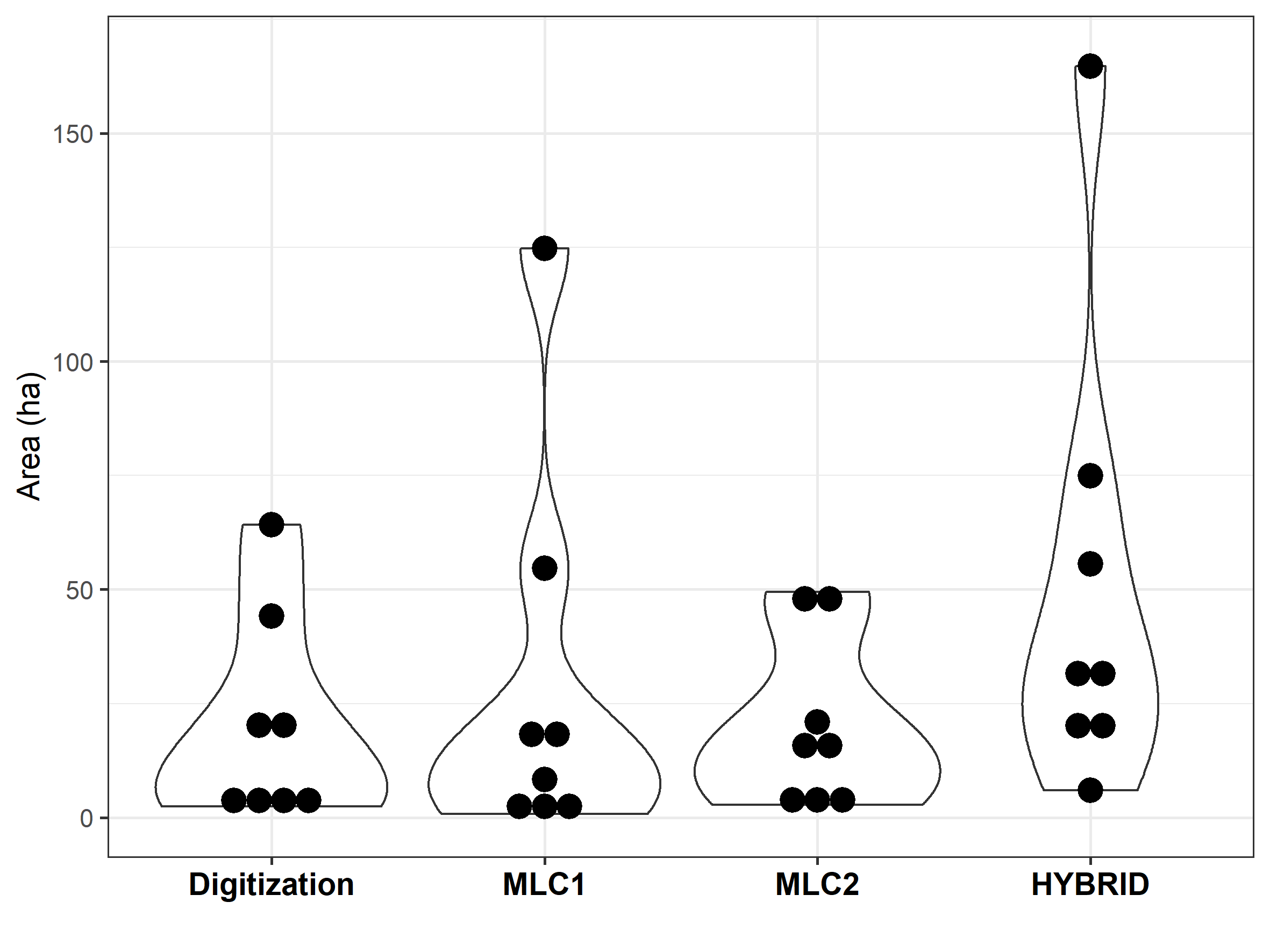

Supplement: S3 Fig — (DOCX) [file pone.0209313.s003.docx]
