# Supplementary material for: Mangroves in the Galapagos islands: Distribution and dynamics
Source: PLoS One. 2019 Jan 9;14(1):e0209313. doi: 10.1371/journal.pone.0209313 (PMC6326481; doi:10.1371/journal.pone.0209313)
Supplement: S4 Fig — Vertical lines represent mean of mangrove patch size for each year. (DOCX) [file pone.0209313.s004.docx]

**S4 Fig. Mangrove patch size (ha) frequency distribution per island and year. Vertical lines represent mean of mangrove patch size for each year.**

**
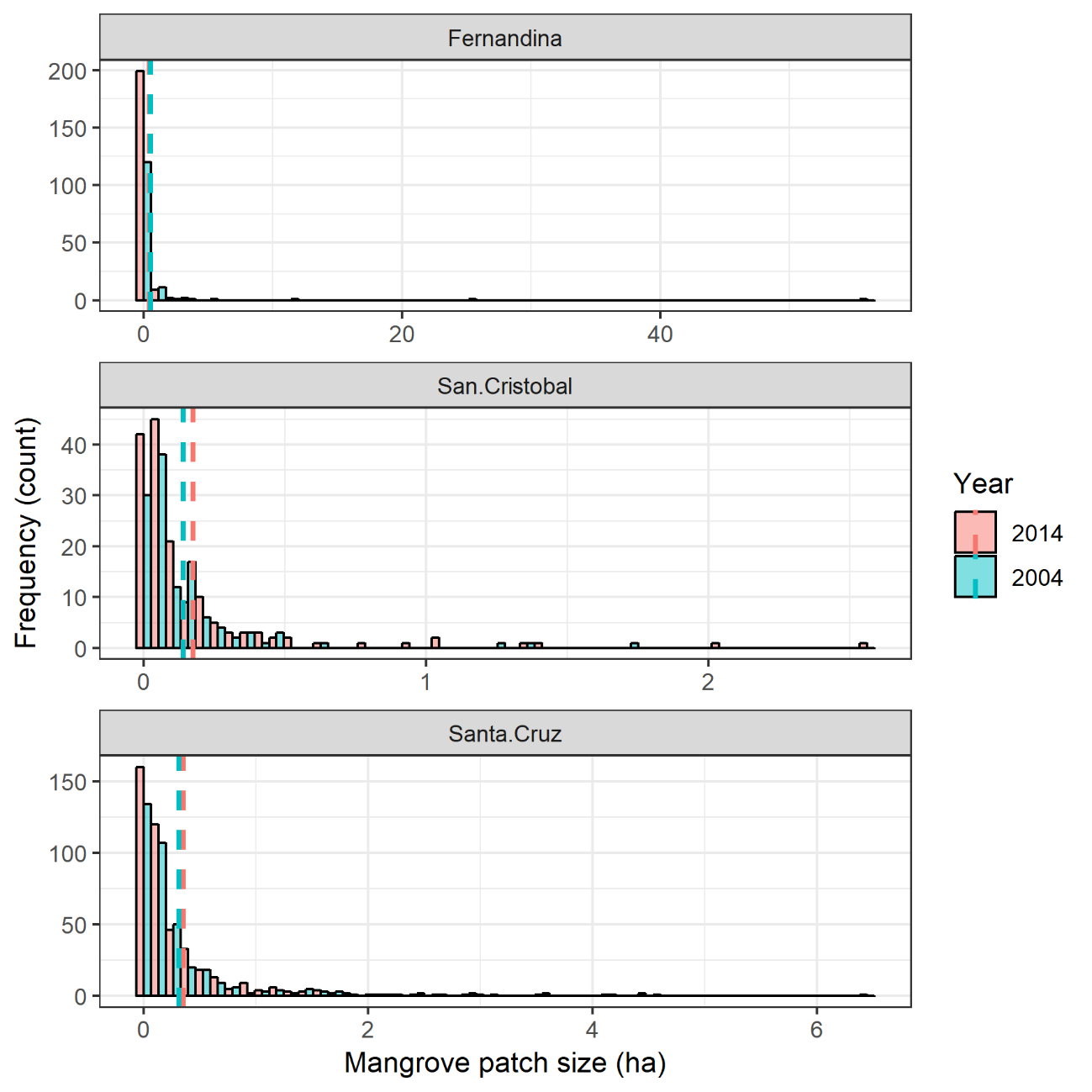
**
