# Supplementary material for: Mangroves in the Galapagos islands: Distribution and dynamics
Source: PLoS One. 2019 Jan 9;14(1):e0209313. doi: 10.1371/journal.pone.0209313 (PMC6326481; doi:10.1371/journal.pone.0209313)

**S5 Fig. Spatial–temporal analysis of moving mangrove patches for the time period 2004-2014 for the three sampled islands (A=Fernandina, B=Santa Cruz, C=San Cristóbal). CONT=contraction, DISA=disappearance, EXPN=expansion, GENR=generation, STBL=stable.** [land shapefile from the Instituto Geográfico Militar, 2013, Base Nacional escala 1:50.000]


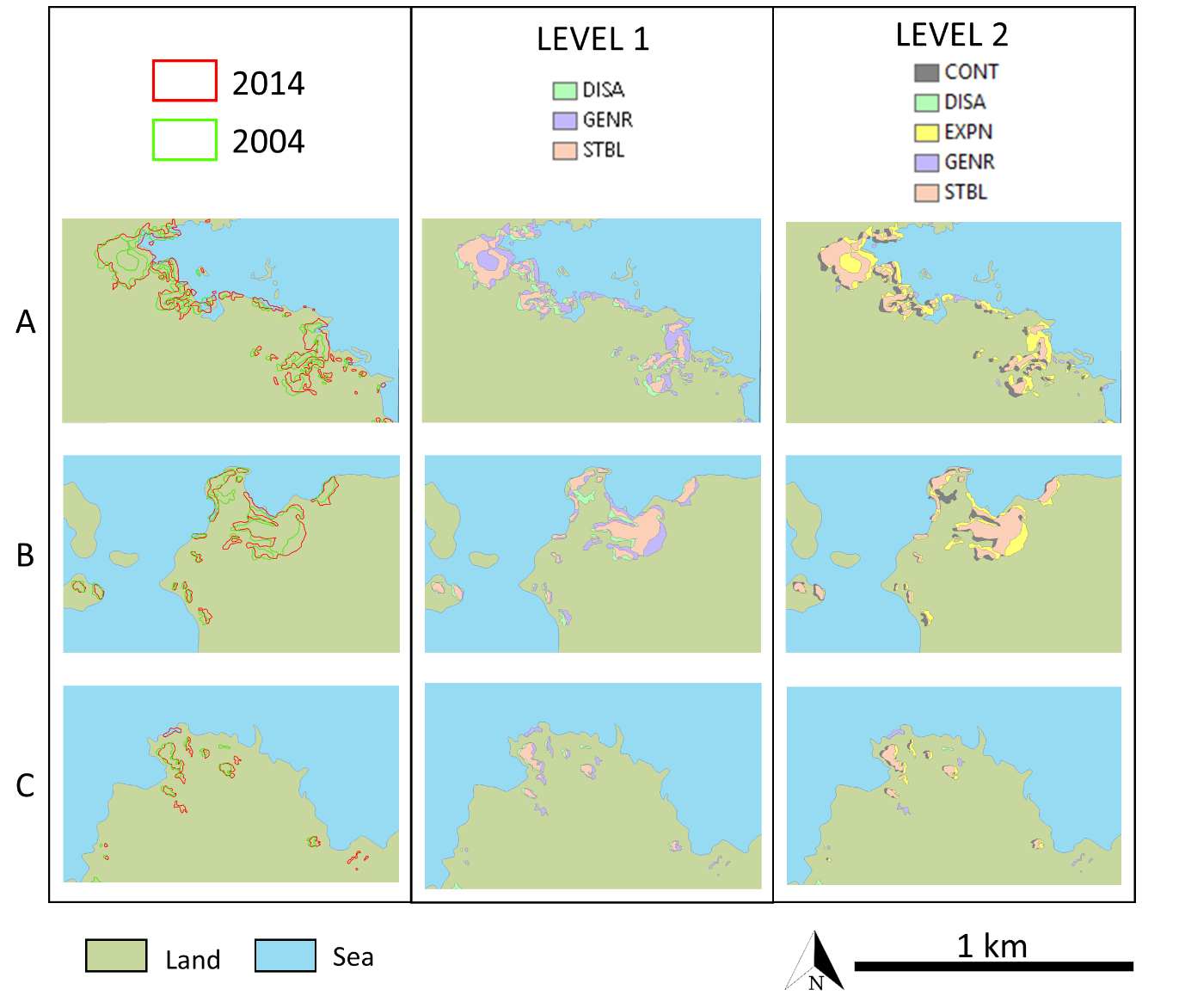

Supplement: S5 Fig — Background screen captured from World Imagery Esri Tile Layer. Locate in: https://services.arcgisonline.com/ArcGIS/rest/services/World_Imagery/MapServer. (DOCX) [file pone.0209313.s005.docx]
