# Supplementary material for: Mangroves in the Galapagos islands: Distribution and dynamics
Source: PLoS One. 2019 Jan 9;14(1):e0209313. doi: 10.1371/journal.pone.0209313 (PMC6326481; doi:10.1371/journal.pone.0209313)
Supplement: S1 Table — (DOCX) [file pone.0209313.s006.docx]

**S1 Table. Google Earth image date and location within the Galapagos Islands used to digitize mangroves in the Galapagos.**

| **ISLAND** | **GOOGLE EARTH IMAGE DATE  format: *mm/dd/yyyy* Location** |
| --- | --- |
| Fernandina | 12/26/2014 E Coastline |
| Isabela | 12/26/2014 NE Coastline (Punta Albermarle) 2/26/2015 E Coastline 08/02/2015 SE (Cuatro Hermanos) 3/19/2014 S 10/21/2011 SW Coastline  12/26/2014 SW Coastline  02/26/2015 W Coastline (Perry’s istmus W) 12/26/2014 NW Coastline 10/15/2003 NW Coastline (Puerto Bravo, Bahía Banks) 11/25/2014 NW Coastline (Oreja del Burro, El Muñeco N) 12/26/2014 N Coastline (Punta Flores) |
| Santiago | 02/06/2014 S Coastline (Poza de las Azules) 02/06/2014 S E Coastline (Sombrero Chino) 02/06/2014 S E Coastline (Rocas Bainbridge) 02/06/2014 E Coastline (Isla Bartolome) 02/06/2014 E Coastline (Isla Bartolome) 02/06/2014 NE Coastline (Cousins) 02/06/2014 NE Coastline (between La Bomba and Punta Cordova) 10/19/2012 N Coastline (W Punta Cordova) 10/19/2012 W Coastline (Caleta Bucanero) 03/1)/2014 W Coastline (Punta Baquerizo) 03/1)/2014 S W Coastline (Puerto Nuevo and Ladilla) 10/19/2012 S Coastline (Cabo Nepean) |
| Rábida | 10/19/2012 N Coastline (Laguna Rábida) |
| Pinzón | 05/13/2007 NE Coastline (Isla Onan) |
| Floreana | 04/01/2014 W Coastline (Puerto Velasco Obarra) 04/01/2014 W Coastline (La Botella) 04/01/2014 W Coastline (Puerto Flores) 04/01/2014 W Coastline (Las Bayas) 04/01/2014 W Coastline (Punta Cormorant) 04/01/2014 E Coastline (Isla Champion) 04/01/2014 E Coastline (Las Tablas) 04/01/2014 E Coastline (Las Cuevas) 04/01/2014 Rest of the island |
| Santa Cruz | 03/19/2014 E Coastline (Islas Plaza) 03/06/2015 E Coastline (Punta Rocafuerte) 03/19/2014 S E Coastline (between Saca Calzón and Garrapatero) 03/19/2014 S E Coastline (between Garrapatero and El Chiquero) 03/19/2014 S Coastline (FCD, Puerto Ayora, Los Alemanes) 03/19/2014 S Coastline (Tortuga Bay hasta Islote La Fé) 03/09/2013 S W Coastline (La Torta)  05/13/2007 S W Coastline (Los Manzanillos) 05/13/2007 W Coastline (Las Palmitas) 09/01/2013 W Coastline (S Las Palmas) 09/01/2013 NW Coastline (Cerro Ballena) 09/01/2013 NW Coastline (Islote Edén) 09/01/2013 NW Coastline (Bahía Conway, Cerro Dragón) 09/01/2013 NW Coastline (Punta Bowditch) 09/01/2013 N Coastline (Caleta Tiburon, Venecia) 09/01/2013 N Coastline (Bahía Borrero) 07/23/2013 N Coastline (Caleta Tortuga) 03/19/2014 N Coastline (La Tranca, frente a Baltra) 07/23/2013 N Coastline (Muelle y Canal de Itabaca) 07/23/2013 N Coastline (Punta Carrión) 03/19/2014 N Coastlineor E (San Vicente) 03/19/2014 N Coastlineor E (Monte Verde) |
| Baltra | 07/23/2013 SE Coastline (Canal Itabaca) 07/23/2013 NW Coastline  07/23/2013 W Coastline media 07/23/2013 SW Coastline (old airport runaway) 07/23/2013 corner of SW Coastline |
| Santa Fé | 01/19/2012 Bahía Santa Fé |
| San Cristóbal | 02/24/2014 S W Coastline (Puerto Baquerizo Moreno) 02/24/2014 S W Coastline (Tijeretas) 02/24/2014 W Coastline (Playa del Muerto) 03/27/2014 W Coastline (Puerto Ochoa) 03/27/2014 W Coastline (Islote Lobos) 03/27/2014 W Coastline (Cerro Mundo) 11/24/2014 W Coastline (Varillaso) 03/27/2014 W Coastline (Manglesito) 03/27/2014 W Coastline (Punta Jely) 03/27/2014 W Coastline (W leon dormido) 03/27/2014 W Coastline (Puerto Grande) 06/15/2005 W Coastline (Punta Pununa) 06/15/2005 W Coastline (Bahía Sardina) 05/11/2005 NW Coastline (Las Salinas, Punta Pitt) 05/11/2005 NE Coastline (S Punta Pitt) 05/11/2005 E Coastline (Puerto Las Tablas) 04/08/2003 E Coastline (Colorado) 12/13/2007 E Coastline media (Rosa Blanca) 12/13/2007 E Coastline media (Roca E) 03/12/2011 E Coastline media (Piedra Ahogada) 03/27/2014 SE Coastline (San Francisco) 03/27/2014 SE Coastline (El Pescador) 03/27/2014 S E Coastline (Las Repisas) 03/27/2014 S E Coastline (Montones de Arena) 03/27/2014 S E Coastline (Chorros de Agua dulce) 03/03/2014 S E Coastline (Espinero) 03/27/2014 S Coastline (Matambre) 03/27/2014 S Coastline (Veinte Varas, Las Negritas) 02/24/2014 S Coastline (La Loberia) 02/24/2014 S Coastline (Punta Wreck) 02/24/2014 S Coastline (Tongo Reef) |
| Española | 05/25/2014 W Coastline (Punta Suarez) 05/25/2014 W Coastline (entre Punta Suarez and El manzanillo) 05/25/2014 W Coastline (El Manzanillo) 05/25/2014 W Coastline (between El Manzanillo and Bahía Gardner) 05/25/2014 N Coastline (Bahía Gardner) 05/25/2014 N Coastline (Islote Gardner) 05/25/2014 NE Coastline (El Yate) 05/25/2014 NE Coastline (Punta Cevallos) 05/25/2014 SE Coastline (La Ventana) 05/25/2014 S Coastline (El Trompo) 05/25/2014 S Coastline (Los Barrancos) 05/25/2014 S Coastline (Soplador) |
