# Supplementary material for: Mangroves in the Galapagos islands: Distribution and dynamics
Source: PLoS One. 2019 Jan 9;14(1):e0209313. doi: 10.1371/journal.pone.0209313 (PMC6326481; doi:10.1371/journal.pone.0209313)
Supplement: S2 Table — MLC1 = classification of the whole image; MLC2 = classification of the land and sea in different phases; HYBRID (OBIA-MLC) = hybrid classification technique consisting of an object based image analysis coupled with MLC. (DOCX) [file pone.0209313.s007.docx]

**S2 Table. Mangrove cover (ha) per method, island and sampled section. MLC1 = classification of the whole image; MLC2 = classification of the land and sea in different phases; HYBRID (OBIA-MLC) = hybrid classification technique consisting of an object based image analysis coupled with MLC.**

| **Sampled Section** | **Island** | **On-Screen Digitization** | **MLC1** | **MLC2** | **HYBRID** |
| --- | --- | --- | --- | --- | --- |
| S1 | Fernandina | 22.0 | 17.4 | 21.0 | 32.4 |
| S2 | Fernandina | 64.2 | 19.0 | 46.4 | 74.9 |
| S3 | Fernandina | 18.5 | 8.3 | 17.9 | 30.7 |
| S4 | Santa Cruz | 44.2 | 124.8 | 49.4 | 164.7 |
| S5 | Santa Cruz | 2.4 | 54.6 | 13.7 | 55.6 |
| S6 | San Cristobal | 5.1 | 1.2 | 2.8 | 18.2 |
| S7 | San Cristobal | 4.0 | 0.8 | 4.3 | 6.0 |
| S8 | San Cristobal | 2.9 | 4.0 | 5.0 | 22.0 |
